# The Interaction Dynamics of Two Potato Leafroll Virus Movement Proteins Affects Their Localization to the Outer Membranes of Mitochondria and Plastids

**DOI:** 10.3390/v10110585

**Published:** 2018-10-26

**Authors:** Stacy L. DeBlasio, Yi Xu, Richard S. Johnson, Ana Rita Rebelo, Michael J. MacCoss, Stewart M. Gray, Michelle Heck

**Affiliations:** 1 United States Department of Agriculture, Biological Integrated Pest Management Research Unit, Robert W. Holley Center for Agriculture and Health, 538 Tower Road, Ithaca, NY 14853, USA; sld98@cornell.edu (S.L.D.); smg3@cornell.edu (S.M.G.); 2 Boyce Thompson Institute for Plant Research, Ithaca, NY 14853, USA; ar973@cornell.edu; 3 Section of Plant Pathology and Plant-Microbe Biology, School of Integrated Plant Science, Cornell University, Ithaca, NY 14853, USA; yx325@cornell.edu; 4 Department of Genome Sciences, University of Washington, Seattle WA 98109, USA; rj8@uw.edu (R.S.J.); maccoss@uw.edu (M.J.M.)

**Keywords:** *Potato leafroll virus*, plant pathogen, phloem-limited, movement protein, viral trafficking, *Luteoviridae*, polerovirus, insect-borne, endomembrane system

## Abstract

The *Luteoviridae* is an agriculturally important family of viruses whose replication and transport are restricted to plant phloem. Their genomes encode for four proteins that regulate viral movement. These include two structural proteins that make up the capsid and two non-structural proteins known as P3a and P17. Little is known about how these proteins interact with each other and the host to coordinate virus movement within and between cells. We used quantitative, affinity purification-mass spectrometry to show that the P3a protein of *Potato leafroll virus* complexes with virus and that this interaction is partially dependent on P17. Bimolecular complementation assays (BiFC) were used to validate that P3a and P17 self-interact as well as directly interact with each other. Co-localization with fluorescent-based organelle markers demonstrates that P3a directs P17 to the mitochondrial outer membrane while P17 regulates the localization of the P3a-P17 heterodimer to plastids. Residues in the C-terminus of P3a were shown to regulate P3a association with host mitochondria by using mutational analysis and also varying BiFC tag orientation. Collectively, our work reveals that the PLRV movement proteins play a game of intracellular hopscotch along host organelles to transport the virus to the cell periphery.

## 1. Introduction

With a limited amount of genetic material, plant viruses have optimized the use of finely-tuned, protein interaction topologies to hijack the cellular machinery of their host and vector to facilitate virus propagation and dispersal [1]. In plant hosts, virus movement occurs in two basic phases: short cell-to-cell movement through micro-channels called plasmodesmata (PD) and long-distance movement through the phloem sieve elements. Both types of movement are facilitated by viral-encoded movement proteins (MPs) [2]. Within cells, viruses exploit host cell trafficking pathways to reach the cell wall where MPs can then ferry virions through PD. Thus, virus-host interactions with viral MPs offer an attractive target for controlling the spread of infection. Characterization of these interactions also provides us with a critical window into how plant cells communicate with one another. There is an abundance of published studies characterizing the activity and intracellular pathways of viral MPs [3,4,5], most notably for the 30-kilodalton movement protein of *Tobacco mosaic virus* (TMV, *Tobamovirus*) and the triple gene block module of potexviruses (e.g., *Potato virus X*). However, information on interactions with host proteins is generally lacking. This is particularly true for the aphid-borne, phloem-limited viruses in the *Luteroviridae* (luteovirids) [6].

Luteovirids are single-stranded, positive-sense RNA viruses that replicate within the cytoplasm of phloem companion and parenchyma cells [7]. Formation of virions is absolutely required for long-distance movement, which indicates a role for the capsid proteins in viral movement [8,9,10,11]. These viruses have a non-enveloped, icosahedral shaped capsid composed of two structural proteins. The coat protein (CP) forms the majority of the 180 protein monomers in the capsid, while the readthrough protein (RTP) comprises a minor yet unknown number of the monomer units. The RTP is generated from the readthrough of a leaky stop codon at the end of the CP open-reading frame, which creates a readthrough domain (RTD) that extends from the surface of the virion. Truncation or amino acid substitutions that alter the C-terminal half of the RTD result in delays in systemic movement and symptom development in the plant [12,13,14,15,16] while substitutions in the N-terminal region of the RTD affect insect transmission [16,17].

In addition to the structural proteins, luteovirids express two non-structural proteins known as P3a and P17 that facilitate long-distance movement of virions *in planta* [18,19]. P17, which is encoded by open reading frame (ORF) 4, shares general characteristics with the MP of TMV [4] in that fluorescent protein fusions of full-length PLRV P17 localize to and increase the size exclusion limit of PD when ectopically expressed [20,21]. PD localization is dependent on several factors including site-specific phosphorylation of the P17 protein sequence [22,23], domains located in the N-terminal and C-terminal regions of the protein [22] and interactions with the actin cytoskeleton and the ER-Golgi secretion system of the host [21]. Dimerization of P17, which is mediated by an amphipathic α-helix domain in the N-terminus of the protein, has been observed by Western blot analysis of PLRV-infected leaf material [24] even though the functional nature of this dimerization has yet to be determined. Similar to other MPs, P17 can also bind single-stranded RNA [25] even though long-distance transport of luteovirids is virion-dependent and cell-to-cell transport of viral ribonucleoproteins has not been reported [8,9,10,11]. Mutational analysis has shown that, in the context of PLRV infection, the absolute requirement for P17 in systemic movement is host-dependent [18].

The recently discovered P3a protein [19] is translated from a non-canonical start codon in ORF3a located immediately upstream of the CP ORF3 in subgenomic RNA1 [19]. This viral protein is small in size (4.8-5.3 kDa), highly conserved among divergent luteovirid species, and contains a putative transmembrane domain at its N-terminus. Null mutants of P3a [19] or a non-synonymous substitution of the conserved proline 18 residue [26] in *Turnip yellows virus* (TuYV, *Polerovirus*) or *Brassica yellows virus* (BrYV, *Polerovirus*), respectively, reduced or abolished the long-distance transport of virions. When fluorescent protein tags were fused to the C-termini of TuYV and BrYV P3a and transiently expressed in *Nicotiana benthamiana* epidermal cells, the fusion proteins localized to the ER, Golgi, and near PD [19,26]. For BrYV, these localization patterns were not affected by substitution of proline 18. In addition, homodimerization of BrYV P3a could also be observed *in planta* [26]. Ectopic expression of P3a proteins from different luteovirid species including the P3a protein from PLRV complimented the systemic movement of a BrYV P3a-null mutant indicating that the movement function of this protein is conserved within the family. Interactions among P3a, P17, virions, and host components are not known and are the focus of this study.

Previously, we used affinity purification coupled to high-resolution mass spectrometry (AP-MS) analysis to identify three nonstructural viral proteins *in complex* with wild type (WT) PLRV that were isolated from locally infected *N. benthamiana* leaves. These included the P1 polyprotein (P1) involved in replication, the RNA-dependent RNA polymerase (RdRP), and the P17 movement protein [27,28]. These publications preceded the discovery of P3a [19]. Therefore, it was not known if P3a would be part of the complex. In this scenario, we extend our *in planta* analysis of the PLRV interactome by using quantitative AP-MS [28] coupled to live cell imaging to compare the levels of P3a co-isolating with WT PLRV and with PLRV mutants defective in the expression of viral proteins and domains associated with PLRV movement. This forward genetics approach reveals that P3a and P17 interact to use multiple organelle membranes to traffic within plants cells and that different domains within P3a and P17 regulate these associations.

## 2. Materials and Methods 

### 2.1. Plant Growth Conditions and PLRV Infection

*N. benthamiana* plants were grown in a growth chamber at 23 °C under 16 h days (light intensity = 150–200 μmoles⋅m^−2^⋅s^−1^) and fertilized (Excel Cal-Mag 15-5-15 at 200 ppm N) once a week. For AP-MS experiments, infiltration of *A. tumefaciens* (LB4404) harboring infectious clones of WT PLRV or PLRV mutants was performed as described in Reference [27]. Mutants tested included the ΔCP mutant that only expresses the non-incorporated form of the RTP due to a deletion of the leaky stop codon between ORF3 and ORF5 [10], a P17-null mutant (ΔP17) that does not express the movement protein [18], the ΔRTD mutant, which expresses CP but lacks the RTD [15,16,28,29], and a ΔRTC mutant (referred to as RTC-3 in Reference [30]) where the last 218 C-terminal residues of the RTD are truncated. All mutants except ΔCP can assemble virions and all exhibit varying degrees of host-dependent delays in systemic movement [15,16,18,30]. Mock infiltrated tissue was used as a negative control and three biological replicate APs with two MS injection replicates were analyzed [31] for each PLRV infection condition. Locally infected tissue was collected three days post infiltration (dpi) and stored at −80 °C until used.

### 2.2. Affinity Purification (AP) of PLRV and Mass Spectrometry

AP was performed as described in Reference [28] using the α-PLRV antibody described in Reference [31]. Isolated PLRV protein complexes were subjected to on-bead reduction, cysteine blocking, and trypsin digestion following the protocol described in Reference [27]. Biological replicates represent an individual set of WT PLRV, mutant PLRV, and mock-infected tissue that were infiltrated, affinity purified, and prepared for MS analysis. The samples were analyzed using the mass spectrometry parameters described in Reference [31].

### 2.3. MS Protein Identification and Label-Free Quantification

Parameters for protein identification are described in Reference [31] using an updated version of our in-house *N. benthamiana-Luteoviridae*-contaminant protein database [28] that included a six-frame translation of PLRV ORF3a. Search results were imported into Scaffold-Q+ version 4.8.4 (Proteome Software, Portland, OR, USA) for label-free quantification by spectral-counting using the protein-clustering feature with the following parameters: a two-peptide minimum, peptide identification threshold ≥90%, and protein identification threshold ≥80%. The false discovery rate was less than 1% on both the protein and peptide level. The relative levels of PLRV CP [32], P3a (YP_009179365.2), P17 (Genbank: NP_056750.1), P1 polyprotein (NP_056747.1), Protein A (AAA56730.1), and IgG (ABD64612.1) were measured across the different AP conditions using Skyline 64-bit version 4.1.0.11714 [33] through integration of the precursor ion (MS1) intensity peak areas for protein specific peptides (Supplemental Table S1). For quantification of protein abundance, the integrated peak areas minus the background (minimum intensity at peak boundaries) for three precursor isotope ions (M, M + 1, and M + 2) were first added to get an abundance value for each peptide than values for each peptide in a protein were averaged together to get a protein abundance value (Supplemental Table S1). Spectra corresponding to P3a specific peptides were manually verified using the Protein Prospector MS-Product web tool (http://propspector.ucsf.edu).

A Shapiro-Wilk test and Quantrile-Quantrile (Q-Q) plot analysis was performed on the measured protein abundance levels using the RStudio® version 3.1.3, (http://www.rstudio.com/) to determine normality. Based on these results, a one-way ANOVA followed by Tukey’s honestly significant difference (HSD) post hoc test was performed on the protein abundance levels of Protein A (Shapiro-Wilk *p*-value = 0.301) to determine statistical significance. All other datasets, having a Shapiro-Wilk *p*-value < 0.05, were subjected to a Kruskal-Wallis rank sum test, which was followed by a Conover post-hoc pairwise multiple comparison with a Holm family-wide error rate (FWER) *p*-value adjustment using the webserver program http://astatsa.com/KruskalWallisTest/. Linear regression analysis was done in Excel. Confidence of interaction with PLRV for P3a, P17, and P1 was assessed by using significance analysis of interactome (SAINT) as described in Reference [28]. SAINT probability scores for these viral proteins in each PLRV infection condition compared to the mock AP controls were above 0.9.

### 2.4. Bimolecular Fluorescent Complementation (BiFC) and Co-Localization Assays

Clones where PLRV P3a was fused downstream of the N-terminal half of the enhanced yellow fluorescent protein (nYFP = residues 1–154) or the C-terminal half (cYFP = residues 155–238) were constructed by amplifying the ORF3a coding sequence without its start codon from our WT infectious clone [29] using primers flanked by the Gateway™ *attB* sites. The P3a amplicon and the sequence for monomeric red fluorescent protein (mRFP) [34] were cloned into the BiFC destination vectors described in Reference [35] using Gateway™ technology (Invitrogen, Carlsblad, CA, USA). To construct vectors where viral proteins were fused upstream of nYFP (1–158) or cYFP (159–238), PLRV ORF3a and ORF4 were amplified without a stop codon and cloned into BiFC expression vectors p2YN and p2YC, which is described in Reference [36]. Fusion proteins expressed from these constructs also contained a glycine linker and human influenza hemagglutinin (HA) epitope tag between the PLRV sequences and YFP tags. Constructs with WT and mutant viral sequences tagged with full-length fluorescent proteins were generated by using Gateway™ technology into the binary vectors pEarley 101, 102, or 104 [37]. For our C-terminally tagged constructs, the non-AUG initiation codon of P3a was changed to AUG to ensure optimal expression. P3a mutant sequences were generated by using a combination of traditional and splice overlap PCR [38] with primers containing nucleotide changes for eliminating codons corresponding to the last 16 residues of P3a (ΔCT) or substituting alanine at the indicated residue positions. Conserved residues were identified by aligning the P3a protein sequences of thirty-four luteovirid species deposited in NCBI following the sequence alignment protocol described in Reference [1]. All PCR primer pairs used for cloning in this study are listed in Table S2. Clones were verified by Sanger sequencing.

Expression vectors were transformed into *A. tumefaciens* (GV3101 or GV2260) and were grown overnight in LB containing the appropriate antibiotics and infiltrated into *N. benthamiana* leaves as described in Reference [39]. Clones were mixed equally with each other and/or CFP [40] or mCherry [41] organelle markers to a final OD_600_ ≤ 0.3 or at the OD_600_ specified. Leaf tissue was harvested at three dpi and immediately imaged live, which is described below. Three leaf punches (3 mm diameter) from all leaves imaged were flash-frozen in liquid nitrogen and stored at −80 °C for Western blot analysis.

### 2.5. Confocal Laser Scanning Microscopy

Leaf discs from the infiltrated area were imaged live using a wet mount with MilliQ-filtered H_2_O. Plasmolysis was achieved by replacing the water with 1–2.5 molar sodium chloride (NaCl) solution and cells imaged after 10 to 15 min. Confocal laser scanning microscopy (CLSM) was performed using a Leica TCS SP5 spectral imaging system (Leica Microsystems, Wetzler, Germany). YFP fluorophore excitation was performed by using a 514-nm argon laser (multiline) and emission spectra were collected at 523 to 591 nm with a hybrid detector (HyD). RFP/mCherry was excited with a 561-nm diode-pumped solid-state (DPSS) laser and detected at 594 to 627 nm with HyD. Chloroplast autofluorescence was excited with DPSS or the argon laser and detected at 675 to 736 nm. CFP fluorescence was excited with a 458-nm argon laser and detected at 468 to 496 nm and scanned sequentially from other fluorescent proteins. Mock infiltrated leaves and those infiltrated with one individual fluorescent protein construct were used as negative controls to set imaging parameters to avoid interference from cellular autofluorescence and bleed-through, respectively. Cells were imaged with a 20X/0.70 water immersion objective (HC PL APO CS) using zoom factors in the XY plane and line averaging (2 to 16). The Z-stack projection was taken at 1.71 μm intervals with a total of 10 steps and time-lapse movies were recorded using a line average of 2 or 4 with the time interval indicated. Fluorescence intensity graphs were generated using the LAS AF 2.4.1 software “quantify intensity” tool (Leica Microsystems, Wetzler, Germany) for the selected region of interest. For co-localization experiments, Pearson’s correlation coefficient (PCC) and Manders’ co-localization coefficient using thresholds (tM) [42] for 10 regions of interest displaying “merged” color fluorescence were measured by using the “Co-localization Threshold” tool in Fiji Image J version 2.0.0-rc-65/1.25b. All measurements had a Costes significance test *p*-value ≥ 0.95 using the Coloc 2 plug-in with 30 to 50 iterations. Micrographs and movies presented represent a subset of all data collected. Each BiFC and co-localization assay was repeated three times.

### 2.6. Immunodetection of PLRV and Fusion Proteins

For visualization of the levels of PLRV CP/RTP in AP input samples and verification of fusion protein size in leaves used for microscopy, cryogenically milled tissue [27] was solubilized on ice for 10 min in an SDS lysis buffer (50 mM Tris-HCL, 10% glycerol, 2.5% SDS, 100 μM DTT, 0.5 mM PMSF, and 1:100 dilution of Halt EDTA-free protease inhibitor cocktail) at a concentration of 200 mg tissue/mL of buffer. Sodium dodecyl sulfate polyacrylamide gel electrophoresis and Western blot analysis was performed, which is described in Reference [27] without deviation for the detection of PLRV CP/RTP. HA tag or GFP polyclonal antibodies SG77 (Invitrogen, Carlsblad, CA, USA) and ab6556 (Abcam, Cambridge, UK), respectively, were used to detect the viral fusion proteins. Quantification of the levels of PLRV in tissue locally infected with WT or the ΔP17 infectious clone using a double-antibody sandwich ELISA (DAS-ELISA) was performed, as described in Reference [39].

## 3. Results 

### 3.1. Expression Levels of the Coat Protein are Variable among Different PLRV Mutants

**Figure 1:**
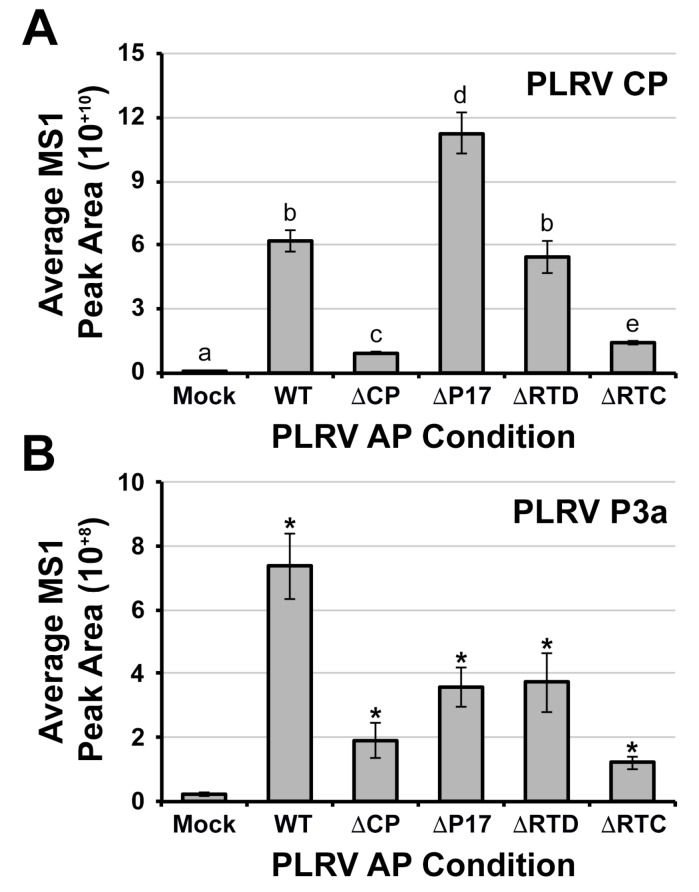
Label-free quantification of PLRV coat protein domain (CP) and P3a in α-PLRV affinity purifications (AP) from leaf tissue infected with WT or PLRV mutants. Bar graphs show the average relative protein abundance of (**A**) CP and (**B**) P3a quantified from the integration of MS1 (precursor ion) peak areas (unit-less) for protein specific peptides detected in AP samples from each of the PLRV infection conditions (*n* = 6 analytical replicates representing three biological replicates). The level of background noise in mock-infected tissue is shown. The number of peptides used for the quantification of protein abundance are CP = 10 and P3a = 2 (Supplemental Table S1). Error bars represent ± the average standard error. Lowercase letters represent significant differences (*p* < 0.05) measured by (**A**) Kruskal-Wallis rank sum test followed by a Conover post-hoc pairwise multiple comparison, which was further adjusted by the Holm FWER method and (**B**) by two-tailed, Wilcoxon signed-rank sum test compared to the mock-infected control where * *p* < 0.05.

We used MS1 peak area integration of peptides specific to the CP domain to quantify the levels of PLRV and/or soluble RTP in each AP sample. We have previously shown that the PLRV antibody is capable of capturing the soluble forms of RTP in addition to assembled virions [27,31]. Although the levels of CP in all PLRV APs were significantly higher than the mock negative controls, we did observe significant differences for some of the PLRV mutants relative to WT PLRV (Figure 1A). Consistent with our previous findings [28], the average levels of CP in the WT and ΔRTD APs were not significantly different (*p* = 0.12, Conover post-hoc test), which indicates an equal enrichment of virion and soluble structural proteins from these two infection conditions. However, the average levels of CP were ~6.5-fold and 4.3-fold lower in ΔCP and ΔRTC APs (*p* < 0.01, Conover post-hoc test) and ~1.8-fold higher in ΔP17 APs (*p* < 0.01, Conover post-hoc test) compared to WT. The levels of protein A and immunoglobulin G (IgG) from beads showed no significant difference across all AP samples including the mock negative controls (Figure S1A,B, *p* > 0.2, Tukey-HSD, and Conover post-hoc test), which indicates that the variation of CP levels across the different infection conditions was not due to technical inconsistency in the amount of beads and/or antibody but rather, due to differences in protein translation efficiency, virus replication or stability in these mutants.

Western analysis of locally infected leaf tissue (Figure S2A) and one representative biological replicate AP (Figure S2B) indicate that levels of CP detected in ΔRTC homogenate and AP eluate were lower relative to WT-PLRV or ΔRTD, which suggests that this mutant is either replication deficient or less stable. As expected, CP was not detected in either the tissue homogenate or the AP eluate of the ΔCP sample (Figure S2A). Interestingly, levels of CP and RTP were higher in the ΔP17 AP compared to WT (Figure S2B). The average virus titer in *N. benthamiana* tissue locally infected with the ΔP17 infectious clone was also significantly higher (~3.5-fold, *p* = 0.003, Student *t*-test) than WT when measured by a double antibody sandwich enzyme-linked immunosorbent assay (Figure S2C). Thus, the higher levels of CP in the ΔP17 AP samples is likely due to an increase in virions in locally infected tissue.

### 3.2. The Viral Protein P3a Complexes with PLRV and the Non-Incorporated Form of the RTP In Vivo

We identified two tryptic peptides corresponding to the C-terminus of P3a in all PLRV AP samples (Figure S3), which enables the quantification of P3a in the viral protein complexes. The peptides R.SIVNEYGR_44_.G (Figure S3A) and R.SIVNEYGRG_45_–(Figure S3B), spanning residues 37–45 (~39% of the total P3a protein sequence) were detected as doubly charged precursor ions with mass to charge ratios of 469.241 *m*/*z* and 497.752 *m*/*z* at an average retention time of 57.7 and 58.1 min, respectively (Supplemental Table S1). The detection of each peptide was confirmed by manual verification of tandem mass spectra associated with each precursor ion (Figure S3). Label-free quantification of P3a protein abundance by averaging the integration of peak areas corresponding to these two precursor ions indicated that P3a was significantly enriched in all PLRV APs compared to mock-infiltrated negative controls (Figure 1B, *p* < 0.05, Wilcoxon signed-rank sum test). SAINT probability scores [43] for P3a were greater than 0.9 in WT and mutant APs compared to mock, which indicated that a high-confidence association with PLRV virion and/or structural proteins [27,28]. These results show that P3a complexes (directly or indirectly) with either assembled virion or free capsid proteins *in planta* but that this association does not require assembly since P3a was significantly enriched in APs from tissue locally infected with the ΔCP mutant (only expresses soluble RTP) compared to mock (Figure 1B).

### 3.3. The Association of P3a with PLRV Is Partially Dependent on the Presence of the P17 Movement Protein

**Figure 2:**
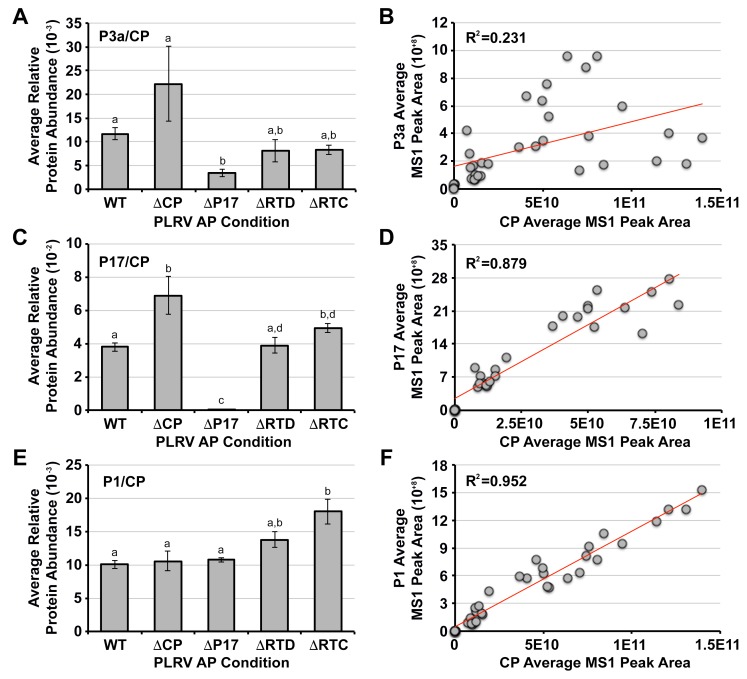
Comparative analysis of WT and mutant PLRV affinity purifications (AP) shows partial loss of P3a when P17 is absent. (**A**,**C**,**E**) Bar graphs show the average, relative protein abundance measured by MS1 peak area integration (unit-less) of (**A**) P3a, (**C**) P17 movement protein, and (**E**) P1 polyprotein in AP samples for each of the PLRV infection conditions normalized to the levels of the PLRV coat protein domain (CP) in those same samples. Error bars represent the ± standard error. Lowercase letters represent a significant difference (*p* < 0.05) measured by a Kruskal-Wallis rank sum test followed by a Conover post-hoc pairwise multiple comparison, which was further adjusted by the Holm FWER method; (**B**,**D**,**F**) Linear regression analysis between the average, relative levels of CP, and (**B**) P3a, (**D**) P17, and (**F**) P1 measured by MS1 peak area integration in each of the analytical AP replicates per PLRV infection condition. The coefficient of determination (R^2^) is given for each regression line (red). Values for P17 and CP in the ΔP17 null-mutant APs were not included in panel (**D**) since P17 was not detected above background noise in these samples (see panel C). The number of peptides used for MS1 peak area integration are: CP = 10, P3a = 2, P17 = 2, and P1 = 4 (Supplemental Table S1).

To determine if the association of P3a with PLRV was dependent on any of the other viral proteins known to participate in movement, we compared the levels of P3a co-immunoprecipitating with each of the PLRV mutants to WT and looked for an increase or decrease of P3a within these samples. Since the amount of captured PLRV varied for the different mutants (Figure 1B), the levels of P3a were normalized to the levels of the CP domain measured in the same sample. These normalized values were then averaged per mutant (Figure 2A). In the ΔCP, ΔRTD, and ΔRTC AP samples, the levels of P3a were similar to WT but were significantly lower in the ΔP17 mutant samples (3.4-fold decrease, *p* < 0.05, Conover post-hoc test, Figure 2A). To assess if the levels of P3a correlated in any way with the variability observed in CP abundance, linear regression analysis was used to compare the levels of these proteins within each mutant and WT PLRV analytical replicate (Figure 2B). This analysis revealed only a weak positive correlation for P3a (R^2^ = 0.231, Figure 2B). In contrast, the levels of P17 (Figure 2C,D) and P1 (Figure 2E,F) [27,28] were found to have a strong positive correlation with the level of the CP domain (R^2^ = 0.879 and 0.952, respectively, Figure 2D,F). As expected, P17 was not detected in APs from tissue infected with the ΔP17 mutant (Figure 2C). However, compared to WT, we did observe a ~1.8-fold increase in the association of P17 with the non-incorporated form of the RTP in the ΔCP mutant (Figure 2B, *p* < 0.001, Conover post-hoc). The levels of P1 were similar between WT PLRV AP samples and all mutants except ΔRTC which exhibited a ~1.8-fold increase compared to WT (*p* < 0.05, Conover post-hoc) (Figure 2C). These data suggest that the interaction of P3a with PLRV is partially dependent on the expression of P17 since abundance of P3a was decreased but not abolished in ΔP17 APs. The interaction (direct or indirect) of both P3a and P17 with virion most likely occurs via the CP domain since these proteins were still enriched in PLRV APs where the RTD was absent (Figure 2, ΔRTD). 

### 3.4. Characterization of PLRV P3a and P17 Homodimerization in Plant Cells 

**Figure 3:**
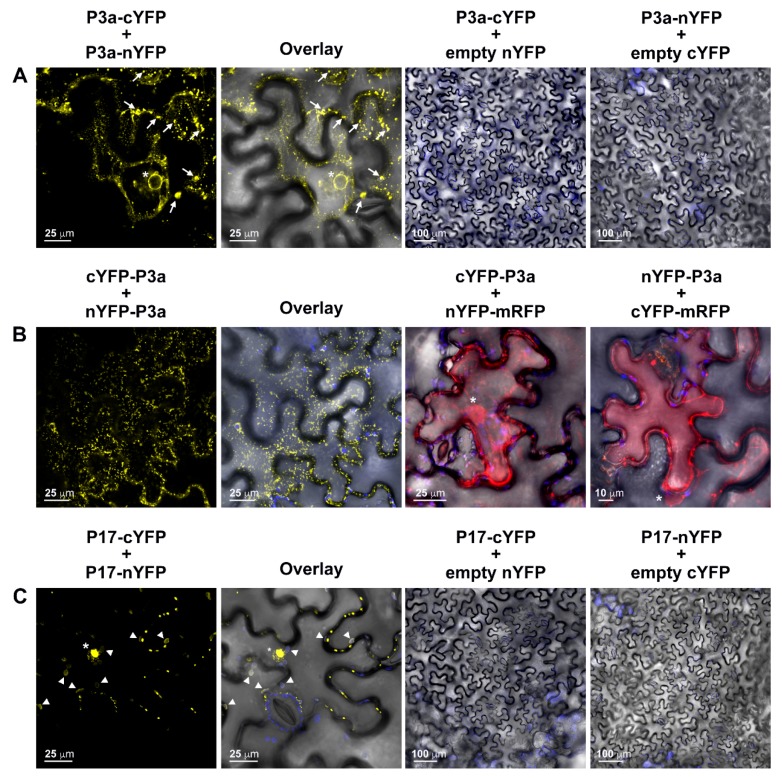
*In planta* self-interaction assays of PLRV P3a shows that tag orientation influences subcellular localization of the P3a homodimer. Panels represent single-plane confocal micrographs of *N. benthamiana* leaf epidermal cells co-expressing bimolecular fluorescence complementation (BiFC) fragments (nYFP and cYFP) fused to the (**A**) C-terminus; (**B**) the N-terminus of P3a; or (**C**) the C-terminus of the PLRV P17 movement protein. The fluorescence signal from the reconstitution of YFP due to self-interaction of the viral proteins is false-colored yellow. Negative controls are represented in the right two micrographs with (**A**) co-expression of the P3a N-terminal BiFC fusions with monomeric red fluorescent protein (mRFP, red fluorescence) fused to the complementary tag and (**B**,**C**) co-expression of the viral C-terminal BiFC fusion proteins with their complimentary empty vector tag. The overlay of the bright field images are shown in the column marked “Overlay” and in negative control images with chloroplast autofluorescence falsely colored blue. Fluorescent protein detection in the nuclei (white asterisks), aggregates (white arrows), and chloroplasts (white arrow heads) are highlighted. Scale bars represent the length indicated in micrometers (μm).

Based on our AP-MS results, we hypothesized that there may be a direct interaction between PLRV P3a and P17. To test this hypothesis, we used bimolecular fluorescence complementation (BiFC) to determine if PLRV P3a and P17 could self-interact and interact with each other *in planta*. Similar to what was reported for BrYV [26], when PLRV P3a was tagged at its C-terminus, the self-interaction was observed as three distinct phenotypes: as tiny, immobile YFP-fluorescent foci (Movie S1), large aggregates within the cytosol, or as a faint “ER-like” network [44] that included the perinuclear space (Figure 3A). When tags were fused to the N-terminus of P3a, an orientation not tested in the BrYV publication [26], self-interaction was observed as highly mobile, dynamic, amorphous spots in the cytoplasm (Figure 3B and Movie S2). This pattern was distinct from the one observed for the C-terminally-tagged homodimer. Consistent with the BrYV study [26], our results show that PLRV P3a also forms dimers in plant cells. However, our analysis also reveals that BiFC tag orientation influences the subcellular localization of the PLRV P3a homodimer. 

In *N. benthamiana* epidermal cells co-expressing P17-cYFP and P17-nYFP, strong YFP fluorescence was observed co-localizing with chloroplast autofluorescence (Figure 3C, Overlay, [PCC = 0.665 ± 0.061, tM_YFP_ = 0.937 ± 0.027 and tM_chloro_= 0.951 ± 0.244]). In addition, YFP fluorescence was observed in several other subcellular locals. These included tiny puncta associated with the nucleus, diffuse fluorescence within the nucleolus, and punctate spots (presumably PD) located along the cell wall. Localization of luteovirid P17 fluorescent protein fusions to PD and nuclei has been previously reported [21,22,45,46]. However, chloroplast localization was only observed when the first 79 to 99 residues of P17 were deleted [22]. Cytoplasmic aggregation of P17 fusion proteins has also been reported [45]. In our hands, expression of PLRV P17 fused to full-length enhanced cyan fluorescent protein (ECFP) at its C-terminus resulted in different subcellular localization patterns depending on the optical density (OD_600_) of the *A. tumefaciens* culture used for infiltration (Figure S4). When bacteria were diluted to an OD_600_ = 0.6, PLRV P17-ECFP localized to cytoplasmic aggregates (Figure S4A, white arrowheads) and filled the nucleus excluding the nucleolus (Figure S4A, white asterisks). When cell density was decreased to OD_600_ = 0.4–0.5, P17-ECFP localized to chloroplasts including stromules (Figure S4B) and puncta associated with the nucleus and cell wall in some cells (Figure S4C) including “vesicle-like” structures surrounding the nucleus that were partially motile (Movie S3). When the culture OD_600_ was ≤0.2, P17-ECFP localized to immobile spots along the cell wall and puncta associated with the nucleus (Figure S4D). The localization patterns observed for P17-ECFP constructs delivered to cells at a lower concentration are similar to the pattern we observed for the P17 BiFC homodimer, which suggests that P17 either traffics as a homodimer to these subcellular compartments and/or self-interacts once there.

### 3.5. P3a Directly Interacts with P17 in Planta

**Figure 4:**
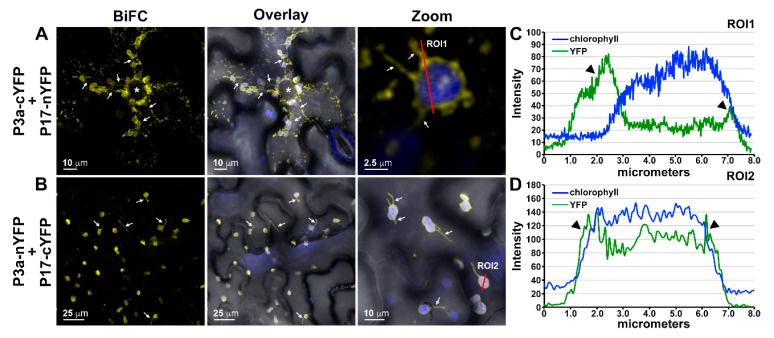
Bimolecular fluorescence complementation (BiFC) assays of PLRV P3a with P17 in leaf epidermal cells show localization of heterodimers to punctate cytoplasmic spots and/or plastids depending on the tag length. (**A**,**B**) Panels represent single-plane confocal micrographs of cells co-expressing (**A**) P3a-cYFP with P17-nYFP and (**B**) P3a-nYFP with P17-cYFP. Reconstitution of YFP due to the interaction of viral proteins is shown as yellow fluorescence. The overlay of BiFC fluorescence with the brightfield view is shown with chloroplast autofluorescence in blue and co-localization of YFP and chloroplast autofluorescence appearing white. The column marked Zoom is a magnification of a selected region within the overlay image. Scale bars show the length indicated. White arrows and asterisks mark chloroplast stromules and nuclei, respectively. (**C**,**D**) Line graphs show the fluorescence intensities of YFP and chlorophyll measured every (**C**) 0.015 and (**D**) 0.072 micrometers across the region of interest (ROI) highlighted by the red line in the Zoom micrographs of panels A and B, respectively. Black arrowheads indicate a region where YFP fluorescence is increased at the edge of the plastid body where chloroplast autofluorescence is in decline.

Using the same C-terminally tagged BiFC constructs and parameters described above, we tested the ability of PLRV P3a to directly interact with P17. In *N. benthamiana* leaf epidermal cells co-expressing P3a fused to the C-terminal half of YFP (cYFP = 9.1 kDa) and P17 to the N-terminal half (nYFP = 17.9 kDa), YFP fluorescence was observed as highly mobile, amorphous spots in the cytoplasm (Figure 4A, movie not shown) similar to the N-terminally tagged P3a homodimer (Figure 3B). Additionally, YFP co-localized with chloroplast autofluorescence (Figure 4A [PCC = 0.509 ± 0.049, tM_YFP_ = 0.931 ± 0.033, tM_chlor_ = 0.945 ± 0.018]) and to stromules [47] (white arrows, Figure 4). When the larger, N-terminal half of split YFP was fused to P3a and co-expressed with P17-cYFP, YFP fluorescence was only observed co-localizing with chloroplast autofluorescence and to plastid stromules (Figure 4B [PCC = 0.788 ± 0.014, tM_YFP_ = 0.968 ± 0.013, tM_chlor_ = 0.974 ± 0.009]). Quantification of fluorescence intensities across individual plastid bodies marked in Figure 4A,B indicated that, as chloroplast autofluorescence from the interior thylakoid membranes decreases at the plastid border, YFP fluorescence was still detected beyond the border especially within the stromule projections (black arrowheads, Figure 4C,D), which suggests localization of P3a-P17 complexes along or within the outer membrane of chloroplasts. These results are in-line with previous ultrastructural studies, which show that PLRV P17 can be detected on the periphery of companion cell chloroplasts in the context of a natural, systemic infection [48]. Unlike the P17 BiFC homodimer (Figure 3C), YFP fluorescence from the P3a-P17 heterodimer interaction was never observed within the nucleus or cell wall (Figure 4A,B). These results suggest that P17 can only localize to the nucleus and the cell wall as a homodimer or that irreversible association with P3a due to BiFC prohibits P17 from trafficking to these sites.

Co-expression of BiFC constructs with P17-ECFP showed that the YFP fluorescent foci of the N-terminally tagged P3a homodimer and P3a-cYFP + P17-nYFP interaction trafficked in proximity to PD-localized P17-ECFP (Figure S5A,B, Movie S4). These mobile viral complexes stalled briefly when a P17-ECFP-labelled PD was encountered (Movie S4). Similarly, P3a-P17 BiFC-labeled plastid membranes were also observed adjacent to P17-ECFP labeled PD (Figure S5B). In some cells, BiFC-labeled stromules extended to the cell periphery from plastids surrounding the nucleus (Figure S5C, white arrowheads) with anterograde and retrograde movement of viral complexes along and/or within stromules (Movie S5,S6). Induction of plasmolysis revealed that, in areas where the plasma membrane receded from the cell wall, BiFC fluorescence was no longer detected near PD-associated P17-ECFP (Figure S5D,E). These data suggest that P3a and P3a-P17 complexes only transiently contact with PD either through the positioning/trafficking of these organelles along the cell wall or possibly through the transport along/within plastid stromules. Localization of these viral complexes within PD either does not occur or does so at a level that is undetected by CLSM.

### 3.6. The P3a Homodimer and P3a-P17 Heterodimer Localize to the Outer Membrane of Mitochondria 

**Figure 5:**
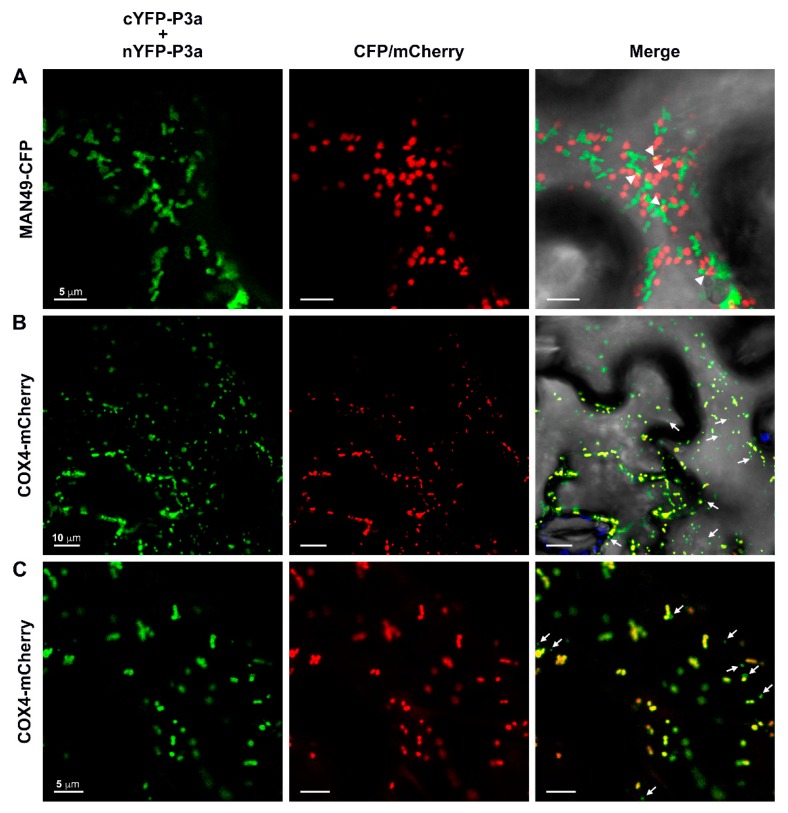
The N-terminally-tagged P3a homodimer co-localizes with mitochondria in *N. benthamiana* leaf epidermal cells. Panels show single-plane confocal micrographs of plant cells co-expressing the bimolecular fluorescence complementation (BiFC) constructs cYFP-P3a plus nYFP-P3a (fluorescence false-colored green) with (**A**) the *cis*-Golgi marker MAN49-CFP or (**B**,**C**) the inner mitochondrial membrane marker COX4-mCherry (red). The overlap of YFP and CFP/mCherry fluorescence with the brightfield overlay image is shown in the column labeled Merge with the co-localization appearing yellow. Chloroplast autofluorescence is falsely colored blue. White arrowheads indicate regions of transient co-localization of the P3a BiFC homodimer and MAN49-CFP. White arrows highlight “vesicle-like” localization of the P3a BiFC homodimer independent of COX4-mCherry fluorescence. Scale bars show the length indicated for each panel.

To identify the subcellular location of the punctate spots observed for the N-terminally tagged P3a BiFC homodimer (Figure 3B) and P3a-cYFP/P17-nYFP heterodimer (Figure 4A), BiFC constructs were co-expressed with fluorescent organelle markers [40,41] in *N. benthamiana* leaves. In contrast to data published for TuYV and BrYV [19,26], widespread co-localization of the YFP signal generated from cYFP-P3a/nYFP-P3a BiFC with MAN49-CFP, a *cis*-Golgi marker [49] was not observed (Figure 5A). Yet, time-lapse analysis did show transient overlap between *cis*-Golgi and the cYFP-P3a/nYFP-P3a BiFC indicating that these two compartments can briefly come in contact (Figure 5A, white arrowheads and Movie S7). We did, however, observe significant co-localization of the cYFP-P3a/nYFP-P3a homodimer (Figure 5B,C [PCC = 0.866 ± 0.021, tMYFP = 0.973 ± 0.008, tMmCherry = 0.954 ± 0.008] and the P3a-cYFP/P17-nYFP heterodimer (Figure S6) with the inner mitochondrial membrane marker, COX4-mCherry, [41]. Interestingly, some of the YFP signal localized to smaller, “vesicle-like” structures, which were devoid of COX4-mCherry (Figure 5B,C, white arrows).

**Figure 6:**
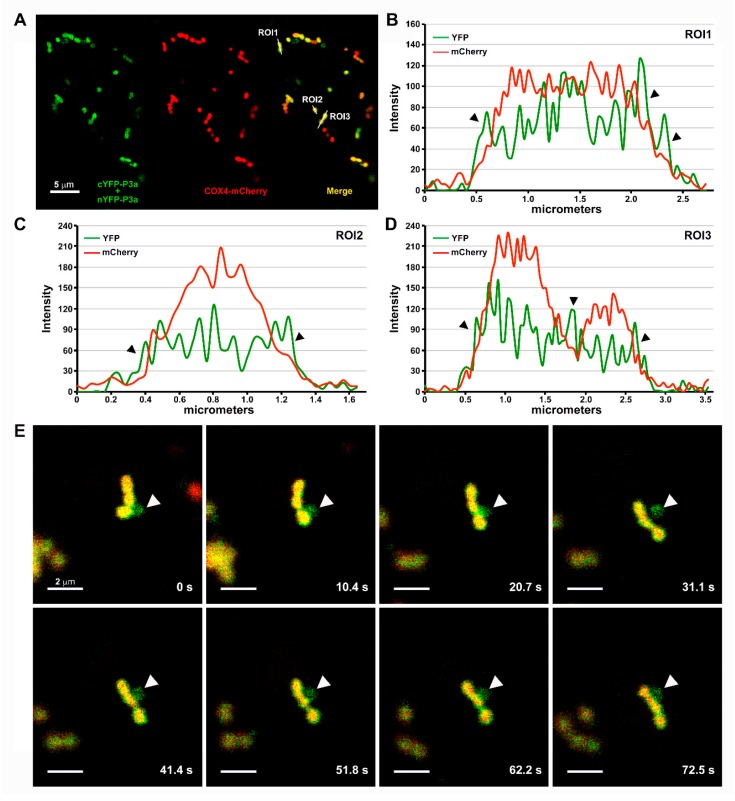
The N-terminally-tagged P3a homodimer localizes to mitochondrial outer membranes and mitochondrial-derived vesicles (MDV). (**A**) A single-plane confocal micrograph montage of a leaf epidermal cell co-expressing the bimolecular fluorescence complementation (BiFC) constructs described in Figure 5; (**B**–**D**) Line graphs show the fluorescence intensities of YFP and mCherry measured every 0.04 micrometers across the region of interests (ROI) highlighted by the white lines in panel A. Black arrowheads indicate a region where YFP fluorescence is increased over mCherry fluorescence at the edges of mitochondria; (**E**) A merged time-course analysis of an *N. benthamiana* leaf epidermal cell co-expressing cYFP-P3a, nYFP-P3a, and COX4-mCherry (red) shows the association and disassociation of a BiFC YFP fluorescent (green) vesicle from a COX4-mCherry labeled mitochondria. Yellow indicates co-localization of YFP and mCherry fluorescence. The panels are representative, single-plane confocal images from Movie S8. The time stamp is in seconds (s).

The quantification of fluorescence intensities across individual mitochondria marked in Figure 6A shows that YFP fluorescence from cYFP-P3a/nYFP-P3a BiFC extended beyond the mitochondrial border marked by the sharp decrease in COX4-mCherry fluorescence (Figure 6B,C, black arrowheads). In the time series presented in Figure 6E (Movie S8), the YFP signal from the N-terminally-tagged P3a homodimer was observed protruding from the body of a mitochondrion marked by COX4-mCherry fluorescence at time point zero. Over time, this protrusion disassociated to form a vesicle (Figure 6E, 31.1s), which re-associated with the mitochondria ~10 s later and proceeded to move up to the tip of the organelle (Figure 6E, 41.4–72.5s). These results demonstrate that some of the YFP-only structures shown in Figure 5A,B (white arrows) and Movie S8 originated from the mitochondrial outer membrane, which is reminiscent of the matrix-free, mitochondrial-derived vesicles (MDVs) that have been observed in plants and animals [50].

**Figure 7:**
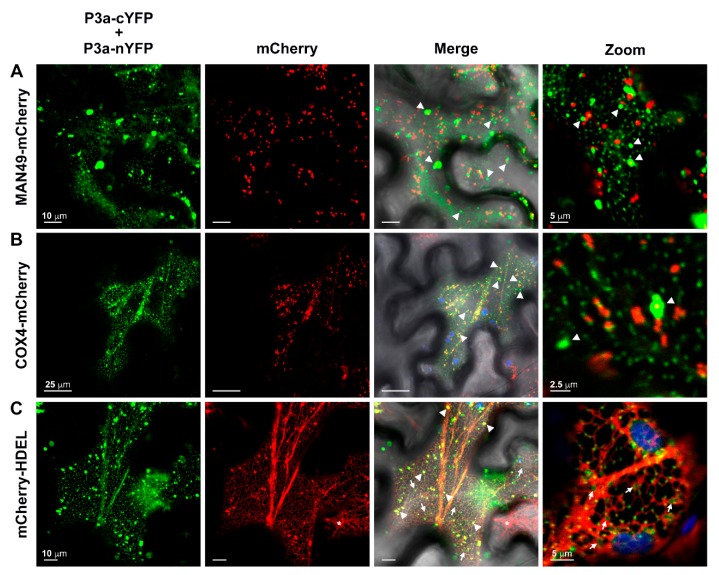
The C-terminally-tagged P3a homodimer localizes to structures associated with the endoplasmic reticulum. The panels represent single-plane confocal micrographs of *N. benthamiana* leaf epidermal cells co-expressing the bimolecular fluorescence complementation (BiFC) constructs P3a-cYFP plus P3a-nYFP with (**A**) the *cis*-Golgi marker MAN49-mCherry; (**B**) the inner mitochondrial membrane marker COX4-mCherry; or (**C**) the mCherry-HDEL, which labels the endoplasmic reticulum (ER) network. The overlap of YFP (green) and mCherry (red) fluorescence images with the corresponding brightfield image is shown in the column labeled merge with co-localization appearing yellow. The column marked Zoom is a magnification of a selected region from the merged image within the same panel. White arrowheads highlight large YFP fluorescent aggregates while arrows mark regions of diffuse YFP single localized in the space between ER tubules. A white asterisk indicates a cell expressing mCherry-HDEL only. Chloroplast autofluorescence is highlighted in blue. Scale bars show the length indicated.

We identified a very different subcellular location for the C-terminally-tagged BiFC P3a homodimer (Figure 3A). This form of the homodimer did not co-localize with *cis*-Golgi (Figure 7A) or mitochondria (Figure 7B) even though significant, transient co-localization could be observed between COX4-mCherry labeled mitochondria and the larger, YFP-fluorescent aggregates, (Figure 7B, [PCC = 0.608 ± 0.030, tM_YFP_ = 0.88 ± 0.020, tM_mCherry_ = 0.917 ± 0.015] and Movie S9). However, YFP fluorescence from the P3a-cYFP/P3a-nYFP interaction did appear as static nodes along the reticulate architecture of the cortical ER, which is highlighted by the organelle marker mCherry-HDEL [41] (Figure 7C, Movie S10). A more diffuse aggregation of BiFC YFP fluorescence was also observed “trapped” within the cytoplasmic space between the ER tubular polygons (Figure 7C, white arrows, Movie S10). The large YFP-fluorescent aggregates also co-localized with mCherry-HDEL (Figure 7C, white arrowheads, [PCC = 0.655 ± 0.031, tM_YFP_ = 0.827 ± 0.029, tM_mCherry_ = 0.884 ± 0.026]), which indicated that these structures may be the result of a local remolding of the ER induced by the expression of the C-terminally tagged P3a homodimer since these aggregates were not observed in an adjacent cell that was only expressing mCherry-HDEL (Figure 7C, white asterisk).

Collectively, our data show that, when the C-terminus of PLRV P3a is unhindered due to tag placement on its N-terminus or a smaller tag at its C-terminus, P3a as a homodimer or *in complex* with P17 can localize within and to the outer membrane of mitochondria including MDVs. If the C-terminus is blocked by the fusion of the larger BiFC tag (nYFP), localization to and/or interaction at the mitochondria is inhibited and P3a-nYFP in the form of a P3a-P17 BiFC heterodimer localizes exclusively to the plastid (Figure 4B) while the C-terminally tagged P3a homodimer localizes to compartments associated with the ER (Figure 7C).

### 3.7. Tag Orientation Influences the Subcellular Localization of PLRV P3a Fused to Full-Length Fluorescent Proteins

To assess the discrepancy in the cellular localization patterns that we observed for PLRV P3a and P3a-P17 in our BiFC assays from the Golgi/ER pattern reported for TuYV and BrYV P3a [19,26], we generated PLRV P3a constructs with the same tag orientation and length used in these previous studies, i.e., full-length ECFP fused to the C-terminus of PLRV P3a (P3a-ECFP). In addition, we generated constructs where full-length EYFP was fused to the N-terminus of PLRV P3a (EYFP-P3a), which is a tag orientation not tested for the other viruses. When *A. tumefaciens* cultures were diluted to an OD_600_ ≤ 0.2, PLRV P3a-ECFP co-localized with the ER in some cells with a stronger fluorescent signal detected at tubular junctions (Figure S7A, white arrows). In other cells, ECFP fluorescence was a mixture of motile, amorphous, and globular-like structures in the cytoplasm (Figure S7A).

**Figure 8:**
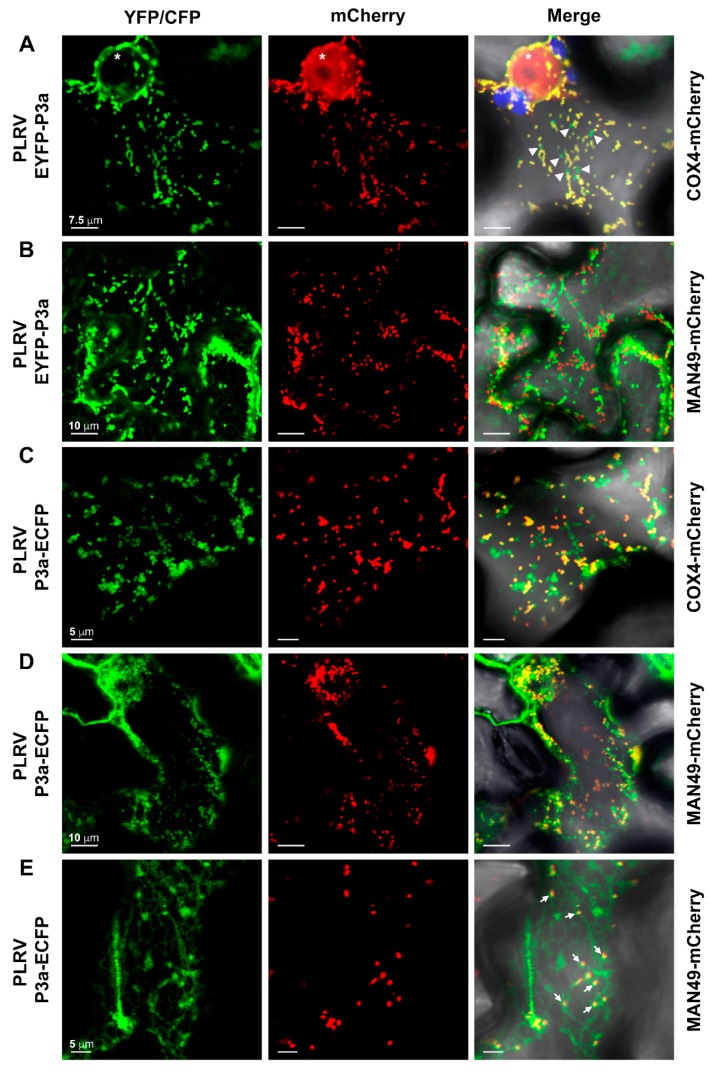
Tag orientation of a full-length fluorescent protein fusion influences the localization pattern of PLRV P3a. The panels represent single-plane confocal micrographs of *N. benthamiana* leaf epidermal cells co-expressing (**A**,**B**) PLRV EYFP-P3a or (**C**,**D**) P3a-ECFP with (**A**,**C**) COX4-mCherry or (**B**,**D**,**E**) MAN49-mCherry. EYFP/ECFP fluorescence is falsely colored green with mCherry fluorescence in red. The overlap of EYFP/ECFP and mCherry fluorescence with the corresponding brightfield image is shown in the column labeled merge with co-localization of fluorescence appearing yellow. Chloroplast autofluorescence is highlighted in blue. White arrowheads indicate EYFP fluorescence independent of COX4-mCherry. A white asterisk marks the position of the nucleus. White arrows mark MAN49-mCherry labeled *cis*-Golgi partially co-localizing with P3a-ECFP at putative ER exit sites. Scale bars show the length indicated.

For EYFP-P3a, we observed faint YFP fluorescence in the network pattern of the ER and strong YFP fluorescence localized to highly mobile spots in the cytoplasm in the same cells (Figure S7B). When the YFP and CFP constructs were co-expressed in the same cell, the ER-like pattern of P3a-ECFP fluorescence became diffuse and the localization to motile, globular, “Golgi-like” structures in the cytoplasm became more defined (Figure S7C). These globular structures remained distinct from the fluorescent pattern of EYFP-P3a (Figure S7C and Movie S11). Co-expression with our organelle markers revealed that EYFP-P3a localized to mitochondria and mitochondrial-derived vesicles (Figure 8A, [PCC = 0.863 ± 0.011, tM_YFP_ = 0.968 ± 0.012, tM_mCherry_ = 0.963 ± 0.007] and Movie S12) but not to *cis*-Golgi (Figure 8B). P3a-ECFP co-localized with both COX4-mCherry (Figure 8C, [PCC = 0.733 ± 0.026, tM_CFP_ = 0.867 ± 0.036, tM_mCherry_ = 0.918 ± 0.013]) and MAN49-mCherry (Figure 8D, [PCC = 0.613 ± 0.042, tM_CFP_ = 0.775 ± 0.053, tM_mCherry_ = 0.833 ± 0.036]). In some cells where the ER-localization pattern of P3a-ECFP was detected, co-localization of the MAN49-mCherry could be observed as cup-like structures along the P3a-ECFP tubular network (Figure 8E, white arrows, [PCC = 0.508 ± 0.029, tM_CFP_ = 0.642 ± 0.040, tM_mCherry_ = 0.701 ± 0.037]). This pattern is similar to the pattern observed for the COPII vesicle coat component Sec16, which localizes to peri-Golgi at ER exit sites [51]. These data support our BiFC results, which show that, PLRV P3a localizes to mitochondria and its outer membrane when its C-terminus is unhindered. However, when a large tag like CFP is located at the C-terminus of the protein, ER to Golgi transport of the PLRV P3a fusion protein occurs.

At the cell periphery, fluorescence from P3a-EYFP was observed in a diffuse pattern along the cell wall (Figure S8A). After plasmolysis, P3a-EYFP-labeled Hechtian strands [52] were observed connecting the retracted plasma membrane to the cell wall (Figure S8B). These strands could also be observed co-localizing with the ER marker mCherry-HDEL (Figure S8C, [PCC = 0.7569 ± 0.054, tM_CFP_ = 0.956 ± 0.012, tM_mCherry_ = 0.947 ± 0.013]), which highlighted the Hechtian reticulum tethered to PD [53]. The C-terminally tagged P3a BiFC homodimer, as static nodes along the cortical ER (Figure 7C), was still observed anchored to the cell wall via the Hechtian reticulum after plasmolysis (Figure S8D). Only a low level of Hechtian strand formation could be observed in plasmolyzed cells co-expressing P17-ECFP with the control plasmid mRFP-YFP (Figure S8E). These results are in contrast, to what we observed for P3a and P3a-P17 complexes that localized to mitochondria and plastids, which did not stay associated with the cell wall after plasmolysis (Figure S5D,E).

### 3.8. Basic Residues in the C-Terminus of PLRV P3a Facilitate Mitochondrial Localization

**Figure 9:**
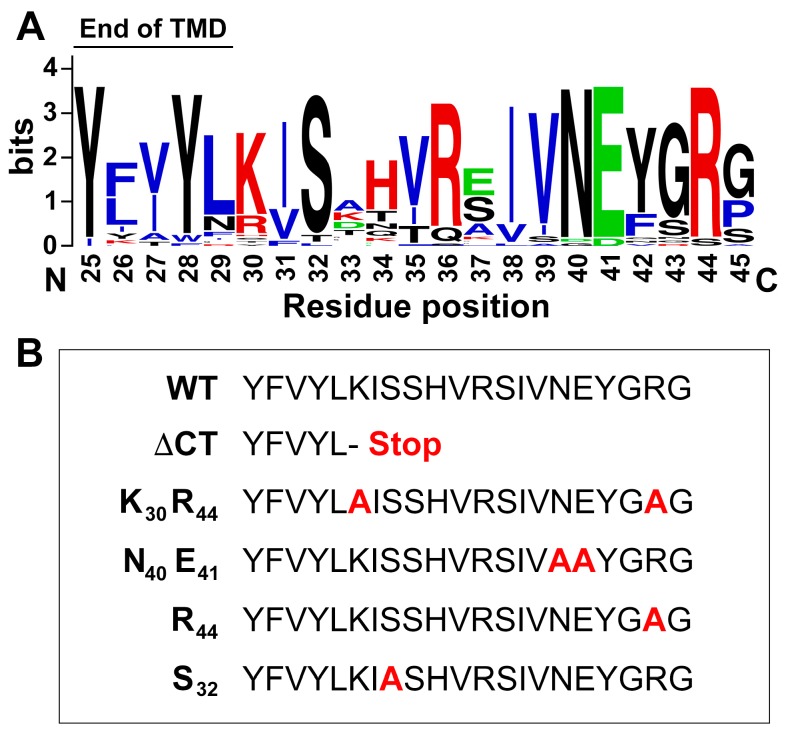

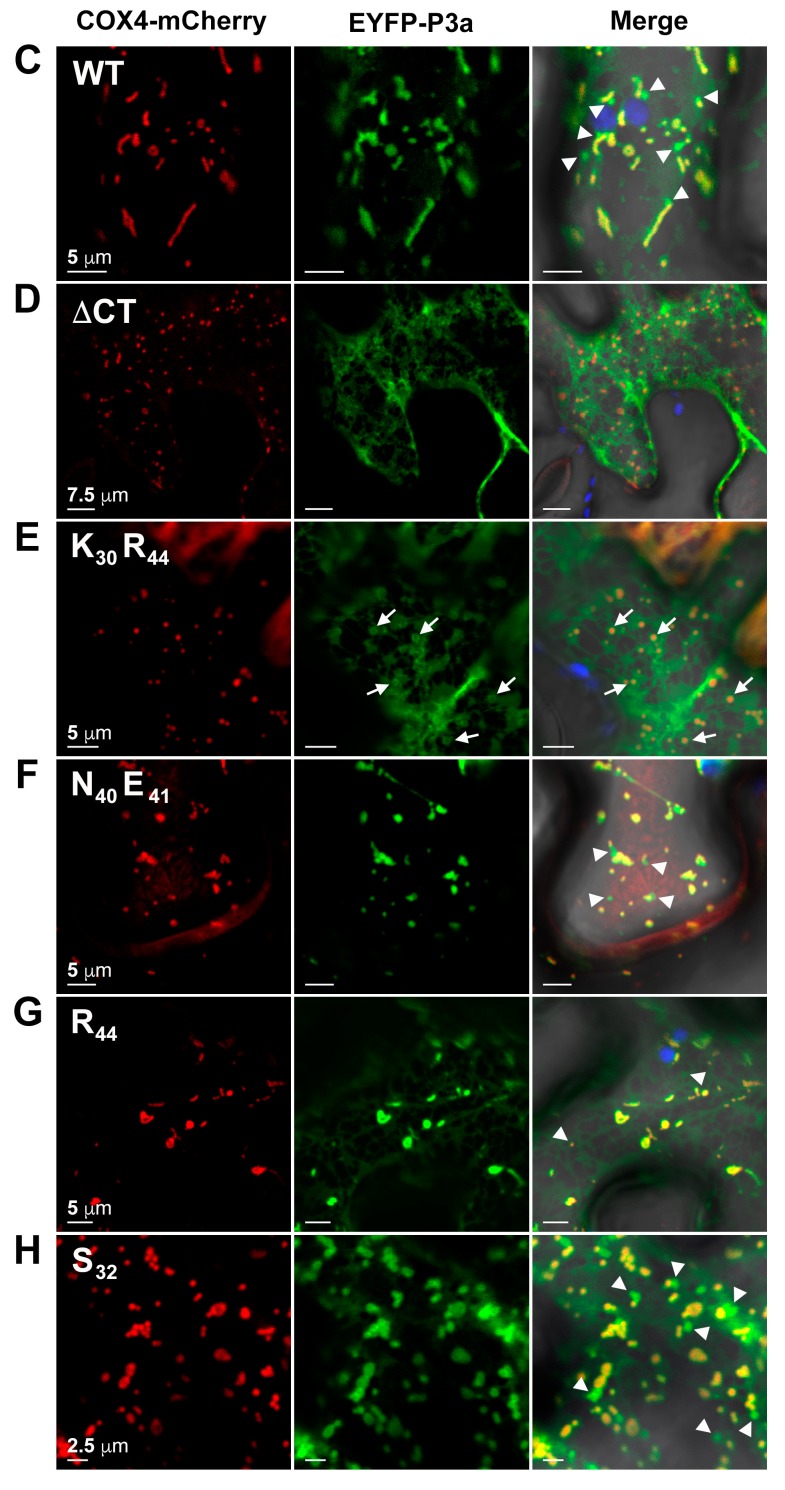
Mutational analysis of the C-terminus of PLRV P3a validates its role in directing mitochondrial localization *in planta*. (**A**) Weblogo alignment of the C-terminal residues of the P3a protein downstream of the predicted transmembrane domain (TMD) from 34 luteovirid species. The height of the stack denotes sequence conservation while the height of the symbol indicates the relative frequency of that amino acid at that position in PLRV P3a. Basic residues are red, acidic in green, and hydrophobic residues in blue; (**B**) Position and identity of the EYFP-P3a C-terminal truncation (ΔCT) and four alanine substitution mutants compared to WT. The amino acid changes are in red and the numbers indicate the amino acid position in PLRV P3a. Only the last five amino acids of the TMD and the 16 residues of the P3a C-terminus in the fusion protein are shown; (**C**–**H**) Panels show single-plane confocal micrographs of *N. benthamiana* leaf epidermal cells co-expressing WT or each of the EYFP-P3a C-terminal mutants (green) with the mitochondrial marker COX4-mcherry (red). The overlap of EYFP and mCherry fluorescence with the corresponding brightfield image is shown in the column labeled Merge with co-localization of fluorescence appearing yellow. Chloroplast autofluorescence is highlighted in blue. White arrowheads indicate MVDs. White arrows label ER-derived ring structures surrounding COX4-mcherry-labeled mitochondria. Scale bars show the length indicated.

Since mitochondrial localization of P3a was only observed when the C-terminus of the viral protein was unhindered by a bulky tag, we hypothesized that a domain in this region may be important for an association with this organelle. The alignment of P3a protein sequences across 34 distinct luteovirid species (poleroviruses and luteoviruses) identified several highly conserved residues in the C-terminus of P3a immediately downstream of the predicted transmembrane domain (Figure 9A). These include three basic residues (lysine, K and arginine, R) at positions 30, 36, and 44 in PLRV P3a, a conserved serine (S) residue at position 32, and two polar residues (asparagine, N and glutamic acid, E) at positions 40 and 41. It has been shown that positively charged residues downstream of hydrophobic domains within membrane-bound proteins promotes their insertion into biological membranes [54]. This peptide region in PLRV P3a has an estimated isoelectric point of pH 10.4 with a net charge of 2.1 at pH 7.0 (https://pepcalc.com/). In order to investigate the role of this C-terminal region in directing the subcellular localization of PLRV P3a, site-directed mutagenesis was used to generate an EYFP-P3a construct deleting the last sixteen residues of P3a immediately downstream of the transmembrane domain and four additional constructs where alanine was substituted for the conserved residues described above either separately or in combination (Figure 9B). These constructs were individually co-expressed in *N. benthamiana* epidermal cells with COX4-mCherry and their localization analyzed by CLSM (Figure 9C–H). Compared to WT EYFP-P3a, which strongly co-localized with COX4-mCherry-labelled mitochondria and to matrix-free MDVs (Figure 9C), truncation of the C-terminus of P3a (ΔCT) abolished co-localization of the fusion protein with COX4-mCherry but not ER localization (Figure 9D). Double substitution of two of the three conserved basic residues in the P3a C-terminus, K_30_ and R_44_ (which lowers the isoelectric point of this region to pH 7.8), also abolished co-localization of EYFP-P3a within COX4-mCherry-labeled mitochondria (Figure 9E). Although, YFP-fluorescent ring structures associated with the ER could be observed surrounding the mitochondria (Figure 9E, white arrows). Substitutions of residues N_40_ and E_41_, R_44_ alone, or S_32_ had no effect on the localization of the EYFP-P3a fusion protein to mitochondria or MVDs (Figure 9F–H). These data further support our BiFC experiments showing that the C-terminus of PLRV P3a, either in conjunction with the predicted N-terminal transmembrane domain or independently, regulates the trafficking of this viral protein to mitochondria.

## 4. Discussion

Previous studies reported that mutations in ORF3a of TuYV or BrYV, which either eliminated expression [19] or created a substitution of a conserved proline residue in the predicted transmembrane domain of the P3a protein [26], nearly abolished the long-distance movement of these viruses in plants (host and non-host). These reports did not establish whether the conserved function of P3a in viral movement was through a direct interaction with virions or indirect by altering the host cellular machinery. In this study, we show that P3a can form protein complexes with PLRV *in planta*, which supports a more direct role for this viral protein in the transport of virions within and between cells. Association of P3a with PLRV was not significantly affected by the loss of virion assembly or soluble CP, since P3a can still complex with the non-assembled form of the RTP. However, we did observe a significant decrease in the association of P3a with PLRV when the P17 movement protein was not expressed. Our results suggest that P3a and P17 work in concert to facilitate the transport of PLRV and/or the non-assembled form of the RTP through their interaction with the CP domain since neither P3a nor P17 was absent in AP experiments where the RTD was deleted or truncated. 

Bimolecular fluorescence complementation assays in *N. benthamiana* epidermal cells confirmed a direct interaction between PLRV P3a and P17 supporting the hypothesis that these two proteins act together to transport viral particles. However, tag sizes and placement on either the N-terminus or C-terminus of P3a influenced the subcellular localization of both the P3a homodimer and P3a-P17 heterodimer species. When tagged at its C-terminus, the PLRV P3a BiFC homodimer localized to immobile, nodes, and aggregates along the ER tubular network. This pattern is similar to what has been reported for synaptotagmin A (SYTA), which is a plant calcium sensor involved in the establishment of ER-PM contact sites that are used by the MPs of tobamoviruses to traffic through PDs [3]. Localization of PLRV P3a to the tubular network of the ER was observed when the protein was tagged with full-length FP on its C-terminus and expressed alone in cells. This pattern was similar to that reported for BrYV P3a [26]. The association of plant RNA viruses and their movement proteins with the ER network of their host is well documented [55] with many using this organelle as a site for replication [56]. PD are plasma membrane lined tubes that are continuous with the cortical ER [2]. Thus, movement through or along the ER offers these plant viruses a direct route to the adjacent cell. For example, transient expression of fluorescent protein fusions of triple gene block movement proteins, TGBp2 and TGBp3, from potexviruses show that these transmembrane domain-containing viral proteins localize to the membrane structures of the ER that are required for systemic movement including virus-induced, vesicles/inclusion bodies near PD [57,58,59,60,61]. We show that both the C-terminally-tagged PLRV P3a homodimer and P3a-ECFP fusion proteins remained associated with the Hechtian reticulum after plasmolysis, which indicates a direct connection with PD [52]. In addition, we observed some remodeling of the ER into large aggregates that co-localized with the C-terminally tagged P3a homodimer. Shepardson et al., (1980) reported that, in PLRV infected phloem cells, virus-induced vesicles could be observed forming from the rough endoplasmic reticulum even though their function was unknown [62]. It is an intriguing possibility that, given the ER localization patterns we observed for the C-terminally-tagged P3a BiFC homodimer and P3a-ECFP, PLRV P3a could be trafficking virions to and through PD via ER-PD junction sites even though luteovirid replication has never been shown to be dependent on the ER like other plant viruses that use this compartment for transport.

We also observed a different subcellular localization pattern for PLRV P3a when BiFC/FP tag placement was on the N-terminus of the viral protein. In this orientation, both the N-terminally-tagged P3a homodimer and EYFP-P3a fusion proteins significantly co-localized with the inner mitochondrial membrane marker COX4-mCherry, the mitochondrial outer membrane, and mitochondrial-derived vesicles. This localization was also observed for the C-terminally tagged P3a-P17 BiFC heterodimer but only when P3a was tagged with the smaller half of YFP. Time-lapse analysis showed that the P3a and P3a-P17-labeled MDVs were dynamic structures that budded from the mitochondrial surface and could be observed trafficking throughout the cell independently, which revealed another putative mode for intra/intercellular transport of PLRV. In both animals and plants, MDVs are produced by the selective incorporation of proteins from either the outer or inner mitochondrial membranes. These vesicles act as carriers of molecules to other organelles [50,63]. Ultrastructural studies have shown that both PLRV virions and P17 can be observed associating with the outer membranes of mitochondria in infected phloem cells including electron-dense globular structures along the organelle border [48,62]. In our study, mitochondrial localization of P17 was only observed when directly bound to P3a-cYFP, which indicates that it is the interaction with P3a during infection that facilitates its association with mitochondria.

Mutational analysis revealed that the ability of P3a to associate with mitochondria is due to structural features in the C-terminus of the protein and not just an artifact of tagging. Truncation of the last 16 residues downstream of the predicted transmembrane domain of PLRV P3a completely inhibited localization of the N-terminally tagged EYFP-P3a fusion protein to mitochondria and MDVs even though localization to the ER was unaffected. The substitution of two highly conserved and positively charged residues to a neutral amino acid resulted in an intermediate phenotype where P3a localized to ER-associated ring structures that surrounded adjacent mitochondria. This pattern could be indicative of incomplete trafficking of the fusion protein from the ER to mitochondria. Substitution of the other highly conserved polar residues or one of the basic residues had no affect. These data suggest that it may be the overall positive charge of the P3a C-terminus, which facilitates association with mitochondria. Cationic sequences are well suited for interacting with negatively charged cellular membranes [54]. This includes most biologically active antimicrobial peptides [64]. It is an intriguing possibility that selection has favored luteovirid proteins to mimic host defense proteins in order to facilitate their association with motile organelles that are bacterial in origin [65].

In addition to mitochondrial localization, our BiFC analysis indicated that, regardless of tag size, the P17 homodimer and P3a-P17 heterodimer localized to plastids where YFP fluorescence was detected within the plastid body and strongly around the outer membrane including stromules, which are stroma-filled tubes generated from the extension of the plastid outer envelope [66]. Since plastid localization of P3a was never observed when any of the P3a fusion proteins were expressed in the absence of P17, these results indicate that once directly bound, P17 can shuttle P3a to the chloroplast membrane. Recently, stromules have been linked to a variety of cellular functions including facilitation of pathogen effector-triggered immunity [47,67,68]. Live imaging of P3a-P17 BiFC labeled stromules revealed that transport of YFP fluorescent spots to and from the cell periphery does occur along these structures. Krenz et al. (2010) demonstrated that the movement protein of *Abutilon mosaic virus*, which is a bipartite geminivirus, directly interacts with a stromule-targeted heat shock protein that, when silenced, partially restricts viral movement [69]. This suggests that stromule formation and/or transport may be a bonafide trafficking route for some plant viruses [70].

## 5. Conclusions

**Figure 10:**
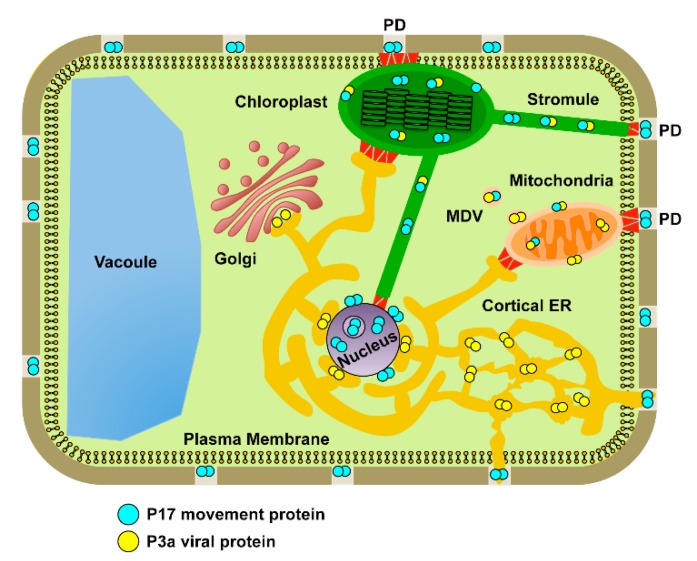
Hypothetical model for the intracellular trafficking of two non-structural viral proteins that regulate long-distance movement of PLRV in plants. Based on imaging results obtained from bimolecular complementation assays coupled to co-localization with fluorescent protein-based organelle markers, P17 (blue circles) and P3a (yellow circles) homo/heterodimer complexes localized to the outer membranes of mitochondria and plastids may traffic to and from plasmodesmata (PD) through plastid stromules, mitochondrial-derived vesicles (MDV), or membrane contact sites (red triangles) established between the cortical endoplasmic reticulum (ER) and other organelles. However, only the P17 movement protein has the ability to permanently associate with PD and the nucleus.

The endomembrane system is a dynamic set of individual compartments that become transiently linked through the formation of organelle projections, vesicles, and/or membrane contact sites that function in the transfer of key signaling molecules and metabolites throughout and between cells. Our results show that P3a and P17 can directly interact *in planta* and that this association facilitates their localization to the outer membranes of mitochondria and plastids including mitochondrial-derived vesicles and plastid stromules with the C-terminus of P3a regulating trafficking to mitochondria. These data suggest that trafficking of PLRV P3a within plant cells is more complex than the ER/Golgi localization pattern that has previously been reported for other luteovirid species [19,26]. It is our hypothesis that PLRV viral movement proteins transport virions and/or structural proteins to the cell periphery by traveling within organelle membranes that transiently come in contact with PD (Figure 10). Our previous characterization of the PLRV-host interactome [27,31] shows that host proteins known to localize to the outer membranes of the nucleus, mitochondria, and plastids form high-confidence interactions with WT PLRV (Table 1) including CHLOROPLAST UNUSUAL POSITIONING 1 (CHUP1), which is a protein involved in the inhibition of stromule formation [68] and promotion of chloroplast translocation to the cell periphery [71]. These viral complexes are most likely transferred from the ER to these various organelles via ER exit-organelle contact sites that are used to traffic endogenous host proteins [44]. Alternatively, but not mutually exclusive, the association of PLRV movement proteins with host organelles that are known regulators of pathogen defense and metabolite biosynthesis may be the mechanism by which PLRV manipulates host physiology to make the cellular environment more favorable for virus replication [39] and/or to attract insect vectors [72]. Further experimentation testing the effects of mutations in the C-terminus of P3a and silencing of host proteins involved in mitochondria/plastid membrane dynamics have on the long-distance movement of luteovirids in host plants are needed to discern between these two possibilities. In addition, the viral fusion proteins generated in this study provide a useful tool to track and capture viral-host protein complexes at distinct subcellular locations for a better understanding of the spatiotemporal interactions that need to occur for successful viral propagation and transmission.

## Figures and Tables

**Table 1:** Proteins identified in the WT PLRV-host interactome that are known to localize to the outer membrane of organelles.

		*N. benthamiana* AP ^c^		*S. tuberosum* AP ^d^	
Organelle ^a^	Host Protein ^b^	Fold Enrichment ^e^	SAINT ^f^	Fold Enrichment ^e^	SAINT ^f^
*Plastid*	TOC75-III	200.6	1	+/−	1
	TOC34	35.3	1	+/−	0.92
	TOC159	33.4	1	nd	nd
	TOC132	+/−	0.8	nd	nd
	OEP16	+/−	0.84	+/−	0.64
	OEP37	+/−	0.82	nd	nd
	CHUP1	+/−	0.85	nd	nd
	HSP12	+/−	0.98	nd	nd
*Mitochondria*	VDAC1	+/−	0.98	1.9	0.61
	DRP1E	10.2	1	7.7	1
*Nucleus*	NOP56	55.7	0.96	+/−	0.64
	NOP5-2	37.8	1	nd	nd
	AT-IMP	53.9	0.93	+/−	0.23
	KPNB1	+/−	0.94	+/−	0.44

^a^ Confirmed membrane localization of host proteins identified *in complex* with PLRV. ^b^ Protein symbol curated from NCBI or Uniprot for *A. thaliana* orthologue by sharing the most significant sequence identity to the *N. benthamiana* and *S. tuberosum* host protein identified *in complex* with WT PLRV. ^c^ Data from the *N.benthamiana*-PLRV interactome reported in Reference [27]. ^d^ Data from the *S. tuberosum*-PLRV interactome reported in Reference [31]. ^e^ Fold enrichment calculation of host protein levels in WT PLRV APs compared to negative controls based on spectral counting. +/− indicates host proteins detected in WT PLRV APs but not negative controls. ^f^ Significance Analysis of INTeractome (SAINT) probability score indicating interaction confidence. nd = not detected.

## Supplementary Materials

The following are available online at http://www.mdpi.com/1999-4915/10/11/585/s1. Figure S1: Bar graphs show the average, relative protein abundance of (**A**) Protein A and (**B**) Immunoglobulin G (IgG) quantified from integration of MS1 (precursor ion) peak areas (unit-less) for protein specific peptides detected in AP samples from each of the PLRV infection conditions (n = 6 analytical replicates representing three biological replicates); Figure S2: Visualization of the levels of PLRV structural proteins in locally infected *N. benthamiana* leaf tissue and a representative set of α-PLRV AP samples used in this study; Figure S3: Representative tandem mass spectra (MS^2^) of doubly-charged tryptic peptides spanning residues 37-45 of the C-terminal end of PLRV P3a; Figure S4: Overexpression of PLRV P17 inhibits its localization to punctate spots along the cell wall and the nucleus; Figure S5: Microscopic observation of plastid and mitochondrial localized P3a-P17 heterodimer complexes at the cell periphery in presence of salt shows association with P17-ECFP along the cell wall is transient; Figure S6: P3a-cYFP/P17-nYFP heterodimer co-localizes with mitochondria in *N. benthamiana* leaf epidermal cells; Figure S7: PLRV P3a localizes to multiple subcellular compartments when fused to a full-length fluorescent protein tag; Figure S8: Plasmolysis of *N. benthamiana* leaf epidermal cells shows localization of PLRV P3a along Hechtian strands and reticulum; Movie S1: The C-terminally tagged PLRV P3a homodimer localizes to motile, punctate spots in the cytoplasm; Movie S2: The N-terminally tagged PLRV P3a homodimer localizes to non-motile, small punctate spots and mobile inclusion bodies in the cytoplasm; Movie S3: The PLRV P17 movement protein fused to ECFP localizes to inclusion bodies associated with the nucleus; Movie S4: Single-plane, CSLM time-lapse imaging of *N. benthamiana* leaf epidermal cells co-expressing cYFP-P3a, nYFP-P3a and P17-ECFP, related to Figure S5A; Movie S5: Single-plane, CSLM time-lapse imaging of a *N. benthamiana* epidermal leaf cell co-expressing P3a-cYFP, P17-nYFP and P17-ECFP, related to Figure S5C; Movie S6: Single-plane, CSLM time-lapse imaging of a *N. benthamiana* epidermal leaf cell co-expressing P3a-cYFP, P17-nYFP and ECFP-HDEL; Movie S7: Co-localization of the C-terminally tagged PLRV P3a homodimer with *cis*-Golgi is minor and transient; Movie S8: The C-terminally tagged PLRV P3a homodimer localizes to mitochondria and mitochondrial derived vesicles; Movie S9: The N-terminally tagged PLRV P3a homodimer transiently co-localizes with mitochondria; Movie S10: Immobile, punctate localization of the N-terminally tagged PLRV P3a homodimer associates with the endoplasmic reticulum tubule network; Movie S11: Tag orientation influences the subcellular localization of PLRV P3a when the viral protein is fused to a full-length fluorescent protein. Movie S12: The YFP-P3a fusion protein localizes to mitochondria. Table S1: Peptides measured by MS1 peak integration. Table S2: Primers used in the construction of BiFC and FP fusion expression clones.
