# Supplementary material for: The Interaction Dynamics of Two Potato Leafroll Virus Movement Proteins Affects Their Localization to the Outer Membranes of Mitochondria and Plastids
Source: Viruses. 2018 Oct 26;10(11):585. doi: 10.3390/v10110585 (PMC6265731; doi:10.3390/v10110585)
Supplement: Supplementary file 1 [file viruses-10-00585-s001.zip › 7.viruses-352914 suppl/viruses-352914 details of suppl.docx]

Supplementary Table S1 Footnotes

^a^NCBI reference number corresponding to protein sequences with peptide spectral matches in our PLRV AP samples.

^b^Protein symbol of PLRV viral proteins and proteins coupled to the surface of magnetic beads that were analyzed in this study.

^c^Amino acid sequence of peptide ions deduced from MS^2^ fragmentation and database searching using Mascot that were used to quantify protein abundance. The residue position of the start and the end of each peptide within the corresponding protein sequence is shown in brackets. The amino acid residues before and after trypsin cleavage are given as a reference. Missed 1 indicates a missed cleavage site. Modified residues are underlined and bold faced.

^d^ Experimentally determined mass to charge ratio of the peptide precursor ion as reported by Mascot.

^e^Average peptide retention time given in minutes.

^f^Charge state of peptide precursor ion analyzed.

^g^Sum of the integrated peak area for all precursor isotope ions ([M]+[M+1]+[M+2]) minus background measured in each analytical replicate AP sample using Skyline MS1 full-scan filtering. Sample names are written as AP biological replicate number analytical replicate number.

Supplementary Table S2 Footnotes

^a^Name of BiFC or FP fusion expression construct generated in this study.

^b^Designated name of primers used for clone construction. F = Forward and R = Reverse primer.

^c^Nucleotide sequence for each primer written from 5’ to 3’. Nucleotides highlighted in bold face correspond to the attB1 and attB2 sequences used for Gateway™ recombination. Nucleotides highlighted in red indicate the added plant Kozak consensus sequence.

Supplemental Figure Legends

**Supplemental Figure S1.** Bar graphs show the average, relative protein abundance of (**A**) Protein A and (**B**) Immunoglobulin G (IgG) quantified from integration of MS1 (precursor ion) peak areas (unit-less) for protein specific peptides detected in affinity purifications (AP) from each of the PLRV infection conditions (n=6 analytical replicates representing three biological replicates). Background noise in mock-infected tissue is shown. The number of peptides used for the quantification of protein abundance are Protein A=8 and IgG=9 (Supplemental Table S1). Error bars represent ± the average standard error. Lowercase letters represent significant difference (*P* < 0.05) measured by (**A**) one-way ANOVA and Tukey’s HSD or **(B**) Kruskal-Wallis rank sum test followed by a Conover post-hoc pairwise multiple comparison, which is further adjusted by the Holm FWER method.

**Supplemental Figure S2.** Visualization of the levels of PLRV structural proteins in locally infected *N. benthamiana* leaf tissue and a representative set of α-PLRV affinity purifications used in this study. (**A-B**) Western analysis using an alkaline phosphatase conjugated, PLRV polyclonal antibody (Agdia) of (**A**) SDS protein extractions from *N. benthamiana* tissue locally infected with WT and mutant PLRV infectious clones, and (**B**) SDS eluates from α-PLRV AP samples (5 mL homogenate/5 mg beads) corresponding to biological replicate one. The levels of the PLRV coat protein (CP, 23.2 kDa, the monomeric, full-length form of WT readthrough protein (RTP, 79.7 kDa), 218 amino acid truncated form of the RTP (RTP*, 55.7 kDa), Rubisco large subunit (RbcL, ~56 kDa, Ponceau stained), and Protein A (Prot A, ~43 kDa) from beads are marked with black arrowheads. Multi-mers of RTP and RTP* with molecular weights <100 kDa are shown but not marked. Tissue and APs from the mock-infiltrated condition (Mock) is shown as a negative control. (**C**) A double-antibody sandwich ELISA using a commercially available α-PLRV capture and detection antibodies (Agdia) shows the relative levels of virion in tissue homogenate (1X PBS) from *N. benthamiana* leaves locally infected with WT PLRV or the ΔP17 mutant clone, which is reported as average Δ Absorbance (450-490 nm). Error bars = ± standard error of n = 5 and 7 leaves (one 3 mm disc per leaf), respectively. ***P* < 0.01 (unpaired, Student T-test).

**Supplemental Figure S3.** Representative tandem mass spectra (MS^2^) of doubly-charged tryptic peptides spanning residues 37-45 of the C-terminal end of PLRV P3a: (**A**) R.SIVNEYGR.G (469.241 *m/z*) and (**B**) R.SIVNEYGRG.- (missed cleavage, 497.752 *m/z*) that were detected and quantified in affinity purifications from PLRV (WT and mutant) infected tissue. Sequence plots showing fragmentation along the peptide backbone indicate the position of *b* and *y* ions used in peptide sequence identification as well as *a* ions produced. RT = peptide retention time; ^++^ = doubly charged ion; ^o^ = loss of H_2_O, ^*^ = loss of NH_3_, MH^+2^ = doubly charged precursor ion. Spectra were produced using collision-induced dissociation (CID) in the linear ion trap mass analyzer.

**Supplemental Figure S4.** Overexpression of PLRV P17 inhibits its localization to punctate spots along the cell wall and the nucleus. (**A-D**) Panels represent single-plane confocal micrographs of *N. benthamiana* leaf epidermal cells three days post-infiltration with decreasing concentrations of *A. tumefaciens* culture carrying a binary plasmid expressing PLRV P17-ECFP driven by a single CaMV 35s promoter. The estimated concentration was measured as the optical density at 600 nm (OD_600_) . Inset in panel (**C**) is a confocal z-stack projection showing the localization pattern of P17-ECFP within and along the nucleus in the region outlined by the white box. ECFP fluorescence is falsely colored red with chloroplast autofluorescence in blue. The overlap of CFP and chloroplast autofluorescence with the brightfield overlay image is shown in the column labeled overlay with co-localization of fluorescence appearing magenta. White asterisks mark position of nuclei. White arrowheads highlight cytoplasmic aggregates of P17-ECFP. White arrows mark sites of P17-CFP localization at the cell wall. Scale bars show the length indicated.

**Supplemental Figure S5.** Microscopic observation of plastid and mitochondrial localized P3a-P17 heterodimer complexes at the cell periphery in the presence of salt shows association with P17-ECFP along the cell wall is transient. The panels represent single-plane confocal micrographs of *N. benthamiana* leaf epidermal cells co-expressing P17-ECFP with bimolecular fluorescence complementation (BiFC) constructs (**A**) cYFP-P3a + nYFP-P3a or (**B-E**) P3a-cYFP + P17-nYFP in the (**A-C**) absence or (**D-E**) presence of 2.5 M salt (+ NaCl). YFP and ECFP are falsely colored green and red, respectively. The overlap of YFP, ECFP, and chloroplast autofluorescence (blue) with the corresponding brightfield image is shown in the column labeled merge. In this column, regions of co-localization appear yellow (YFP with ECFP), magenta (YFP with chloroplast autofluorescence), and light blue (ECFP with chloroplast autofluorescence). White arrowheads indicate chloroplast stromules with the white-dotted line showing the position of the plasma membrane in plasmolyzed cells. Scale bars show the length indicated.

**Supplemental Figure S6.** P3a-cYFP/P17-nYFP heterodimer co-localizes with mitochondria in *N. benthamiana* leaf epidermal cells. Panels show single-plane confocal micrographs of plant cells co-expressing the P3a-cYFP and P17-nYFP constructs (bimolecular fluorescence complementation is false-colored green) with the mitochondrial marker COX4-mCherry (red). The overlap of YFP and mCherry fluorescence with the brightfield overlay image is shown in the column labeled merge with co-localization appearing yellow. Chloroplast autofluorescence is falsely colored blue. Overlap with BiFC fluorescence appears light blue. The panel marked Zoom is a magnification of a selected region of the cell shown in the other panels. A white asterisk denotes the position of the nucleus. Scale bars show the length indicated**.**

**Supplemental Figure S7.** PLRV P3a localizes to multiple subcellular compartments when fused to a full-length fluorescent protein tag. The panels represent single-plane confocal micrographs of *N. benthamiana* leaf epidermal cells expressing (**A**) P3a-ECFP (red), (**B**) EYFP-P3a (green), or (**C**) both constructs. The overlap of EYFP and ECFP fluorescence with the corresponding brightfield image is shown in the column labeled merge with co-localization of fluorescence appearing yellow. The column marked Zoom is a magnification of a selected region from the merged image within the same panel with chloroplast autofluorescence highlighted in blue. White arrows indicate P3a-ECFP localization at tubular junctions. White arrowheads show localization of P3a-ECFP to globular-like structures in the cytoplasm. Scale bars show the length indicated.

**Supplemental Figure S8.** Plasmolysis of *N. benthamiana* leaf epidermal cells shows localization of PLRV P3a along Hechtian strands and reticulum. Panels show representative, single-plane confocal images of plant cells co-expressing (**A-C**) P3a-EYFP, (**D**) P3a-cYFP + P3a-nYFP, or (**E**) mRFP-EYFP with (**A-B, E**) P17-ECFP or (**C-D**) mCherry-HDEL with YFP fluorescence shown in green and CFP/mCherry fluorescence in red. The cells exposed to 1M NaCl are marked by the black bracket labeled + NaCl. The overlap of YFP, ECFP, and chloroplast autofluorescence (blue) with the corresponding brightfield image is shown in the column labeled merge with regions of YFP and CFP/mCherry co-localization appearing yellow. White arrowheads highlight punctate localization of the P3a-cYFP/P3a-nYFP homodimer along the Hechtian reticulum. Scale bars show the length indicated.

Supplemental Movie Legends

**Movie S1.** The C-terminally tagged PLRV P3a homodimer localizes to motile, punctate spots in the cytoplasm. Movie shows single-plane, confocal laser scanning microscopy (CLSM) time-lapse imaging of *N. benthamiana* epidermal leaf cells co-expressing cYFP-P3a and nYFP-P3a related to Figure 3A. The YFP signal due to bimolecular fluorescence complementation (BiFC) is falsely colored yellow with chloroplast autofluorescence highlighted in blue. The cell wall boundaries are shown in the brightfield image overlay. The time series interval was 20.726 seconds. Movie speed is three frames per second (fps) with a total of 15 frames. The scale bar length is indicated.

**Movie S2.** The N-terminally tagged PLRV P3a homodimer localizes to non-motile, small punctate spots and mobile inclusion bodies in the cytoplasm. Movie shows single-plane, CSLM time-lapse imaging of a *N. benthamiana* epidermal leaf cell co-expressing P3a-cYFP and P3a-nYFP constructs related to Figure 3B. The YFP signal due to BiFC is false-colored green with a chloroplast auto-fluorescence highlighted in blue. The cell wall boundaries are shown in the brightfield image overlay. The time series interval was 10.360 seconds. Movie speed is three fps with a total of 20 frames. The scale bar length is indicated.

**Movie S3.** The PLRV P17 movement protein fused to ECFP localizes to inclusion bodies associated with the nucleus. Single-plane, CSLM time-lapse imaging of a *N. benthamiana* epidermal leaf cell expressing P17-ECFP related to Figure S4. Concentration of *A. tumefaciens* solution infiltrated was OD_600_=0.4. Fluorescence from ECFP is falsely colored red with chloroplast autofluorescence in blue. The cell wall boundaries are shown by the overlay of the corresponding brightfield images. The time series interval was 10.360 seconds. Movie speed is three fps with a total of 15 frames. Scale bar length is indicated.

**Movie S4.** Single-plane, CSLM time-lapse imaging of *N. benthamiana* leaf epidermal cells co-expressing cYFP-P3a, nYFP-P3a, and P17-ECFP related to Figure S5A. Movie shows prolonged and transient association of the mitochondrial-localized P3a BiFC homodimer with P17-CFP at the cell wall. Fluorescence from BiFC and ECFP are false-colored green and red, respectively, with chloroplast autofluorescence in blue. The cell wall boundaries are shown in the brightfield image overlay. The time series interval was 10.360 seconds. Movie speed is two fps with a total of 15 frames. The scale bar length is indicated.

**Movie S5.** Single-plane, CSLM time-lapse imaging of a *N. benthamiana* epidermal leaf cell co-expressing P3a-cYFP, P17-nYFP, and P17-ECFP related to Figure S5C. Movie shows BiFC YFP fluorescence (green) associated with and moving along stromules extending to the cell periphery from plastids (blue) clustering around the nucleus. Fluorescence from P17-ECFP is falsely colored red. Overlap of YFP and chloroplast autofluorescence appears light blue. The cell wall boundaries are shown in the brightfield image overlay. The time series interval was 10.360 seconds. Movie speed is three fps with a total of 20 frames. The scale bar length is indicated.

**Movie S6.** Single-plane, CSLM time-lapse imaging of a *N. benthamiana* epidermal leaf cell co-expressing P3a-cYFP, P17-nYFP, and ECFP-HDEL. Movie shows anterograde and retrograde movement of punctate BiFC YFP fluorescence (green) along a plastid stromule extending to the cell periphery. Fluorescence from ECFP-HDEL is falsely colored red. Overlap of YFP and chloroplast autofluorescence appears light blue. Cell wall boundaries are shown in the brightfield image overlay. Time series interval was 10.360 seconds. Movie speed is three fps with a total of 20 frames. The scale bar length is indicated.

**Movie S7.** Co-localization of the C-terminally tagged PLRV P3a homodimer with *cis*-Golgi is minor and transient. Movie shows single-plane, CSLM time-lapse imaging of an *N. benthamiana* epidermal leaf cell co-expressing cYFP-P3a, nYFP-P3a, and the *cis*-Golgi marker MAN49-ECFP related to Figure 5A. YFP signal due to BiFC is falsely colored green with MAN49-ECFP fluorescence in red and chloroplast autofluorescence highlighted in blue. Transient co-localization of YFP and ECFP fluorescence appears yellow. The cell wall boundaries are shown in the brightfield image overlay. Time series interval was 10.386 seconds. Movie speed is three fps with a total of 15 frames. The scale bar length is indicated.

**Movie S8.** The C-terminally tagged PLRV P3a homodimer localizes to mitochondria and mitochondrial derived vesicles. Movie shows single-plane, CSLM time-lapse imaging of a *N. benthamiana* epidermal leaf cell co-expressing cYFP-P3a, nYFP-P3a, and the inner mitochondrial membrane marker COX4-mCherry related to Figure 5C and 5A-H. The YFP signal due to BiFC is falsely colored green with COX4-mCherry fluorescence in red. Co-localization of YFP and mCherry fluorescence appears yellow. The time series interval was 10.360 seconds. The movie speed is two fps with a total of 20 frames. The scale bar length is indicated.

**Movie S9.** The N-terminally tagged PLRV P3a homodimer transiently co-localizes with mitochondria. The movie shows single-plane, CSLM time-lapse imaging of a *N. benthamiana* epidermal leaf cell co-expressing P3a-cYFP, P3a-nYFP, and the inner mitochondrial membrane marker COX4-mCherry related to Figure 7B. YFP signal due to BiFC is falsely colored green with COX4-mCherry fluorescence in red and chloroplast autofluorescence highlighted in blue. Transient co-localization of YFP and mCherry fluorescence appears yellow. The cell wall boundaries are shown in the brightfield image overlay. The time series interval was 10.360 seconds. The movie speed is two fps with a total of 20 frames. The scale bar length is indicated.

**Movie S10.** Immobile, punctate localization of the N-terminally tagged PLRV P3a homodimer associates with the endoplasmic reticulum tubule network. Movie shows single-plane, CSLM time-lapse imaging of a *N. benthamiana* epidermal leaf cell co-expressing P3a-cYFP, P3a-nYFP, and the endoplasmic reticulum marker mCherry-HDEL related to Figure 7C. YFP signal due to BiFC is falsely colored green with mCherry-HDEL fluorescence in red. Transient co-localization of YFP and mCherry fluorescence appears yellow. Two large regions of YFP fluorescence at the top left are chloroplasts. The cell wall boundaries are shown in the brightfield image overlay. The time series interval was 10.360 seconds. The movie speed is two fps with a total of 20 frames. The scale bar length is indicated.

**Movie S11.** Tag orientation influences the subcellular localization of PLRV P3a when the viral protein is fused to a full-length fluorescent protein. The movie shows single-plane, CSLM time-lapse imaging of a *N. benthamiana* epidermal leaf cell co-expressing EYFP-P3a and P3a-ECFP related to Figure S5C. Fluorescence from EYFP and ECFP are falsely colored green and red, respectively, with transient co-localization appearing yellow. The cell wall boundaries are shown in the brightfield image overlay. The time series interval was 15.540 seconds. The movie speed is three fps with a total of 20 frames. The scale bar length is indicated.

**Movie S12.** The YFP-P3a fusion protein localizes to mitochondria. Movie shows single-plane, CSLM time-lapse imaging of a *N. benthamiana* epidermal leaf cell co-expressing EYFP-P3a and the mitochondrial inner membrane marker COX4-mCherry related to Figure 8A. Fluorescence from EYFP and mCherry are falsely colored green and red, respectively, with co-localization appearing yellow. Chloroplast autofluorescence is highlighted in blue and the brightfield image overlay shows the cell wall boundaries. The time series interval was 10.360 seconds. The movie speed is two fps with a total of 20 frames. The scale bar length is indicated.
