# Supplementary figures and images for: The Interaction Dynamics of Two Potato Leafroll Virus Movement Proteins Affects Their Localization to the Outer Membranes of Mitochondria and Plastids
Source: Viruses. 2018 Oct 26;10(11):585. doi: 10.3390/v10110585 (PMC6265731; doi:10.3390/v10110585)

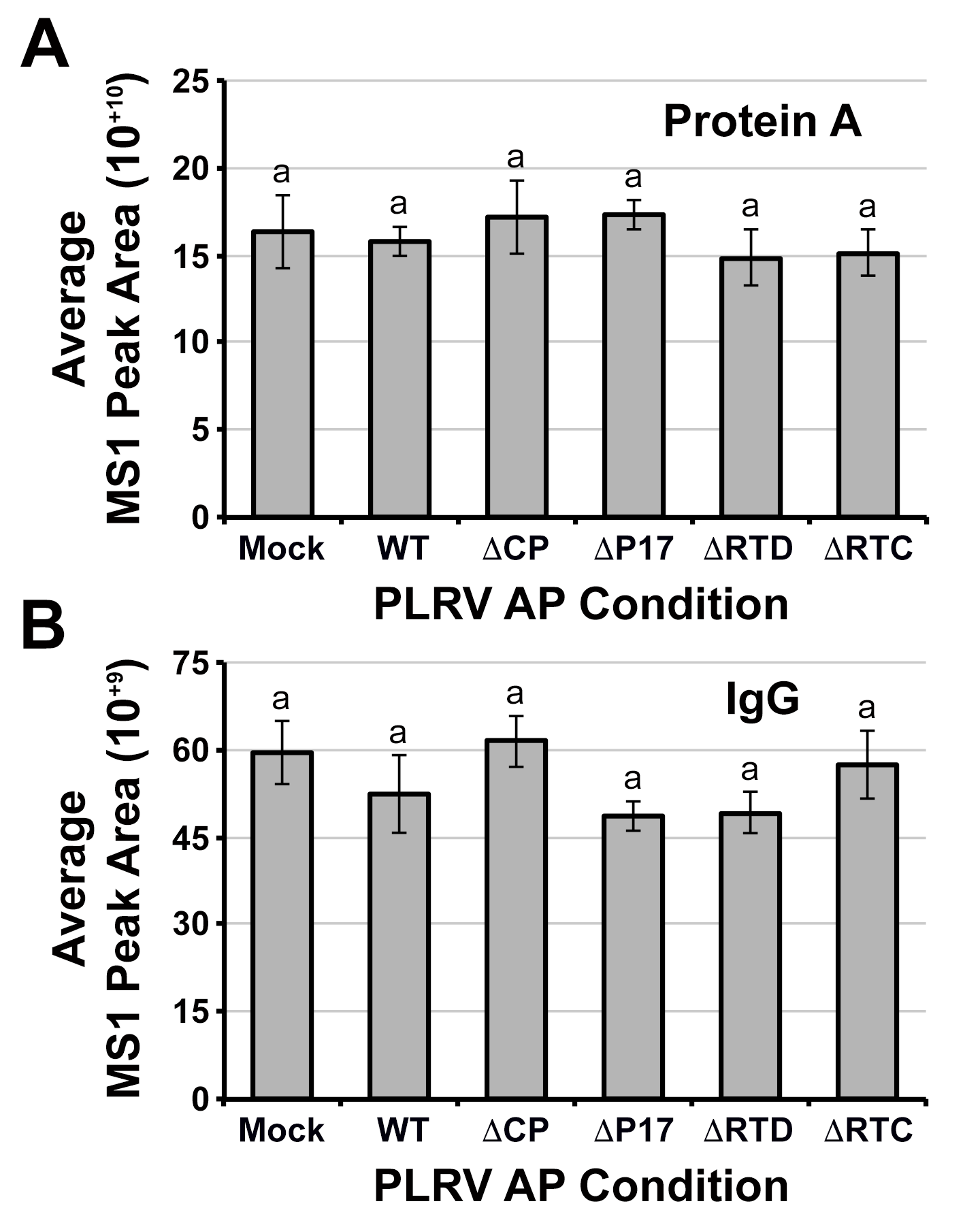

Supplement: Supplementary file 1 [file viruses-10-00585-s001.zip › 7.viruses-352914 suppl/DeBlasio_Viruses_FigureS1.tif]

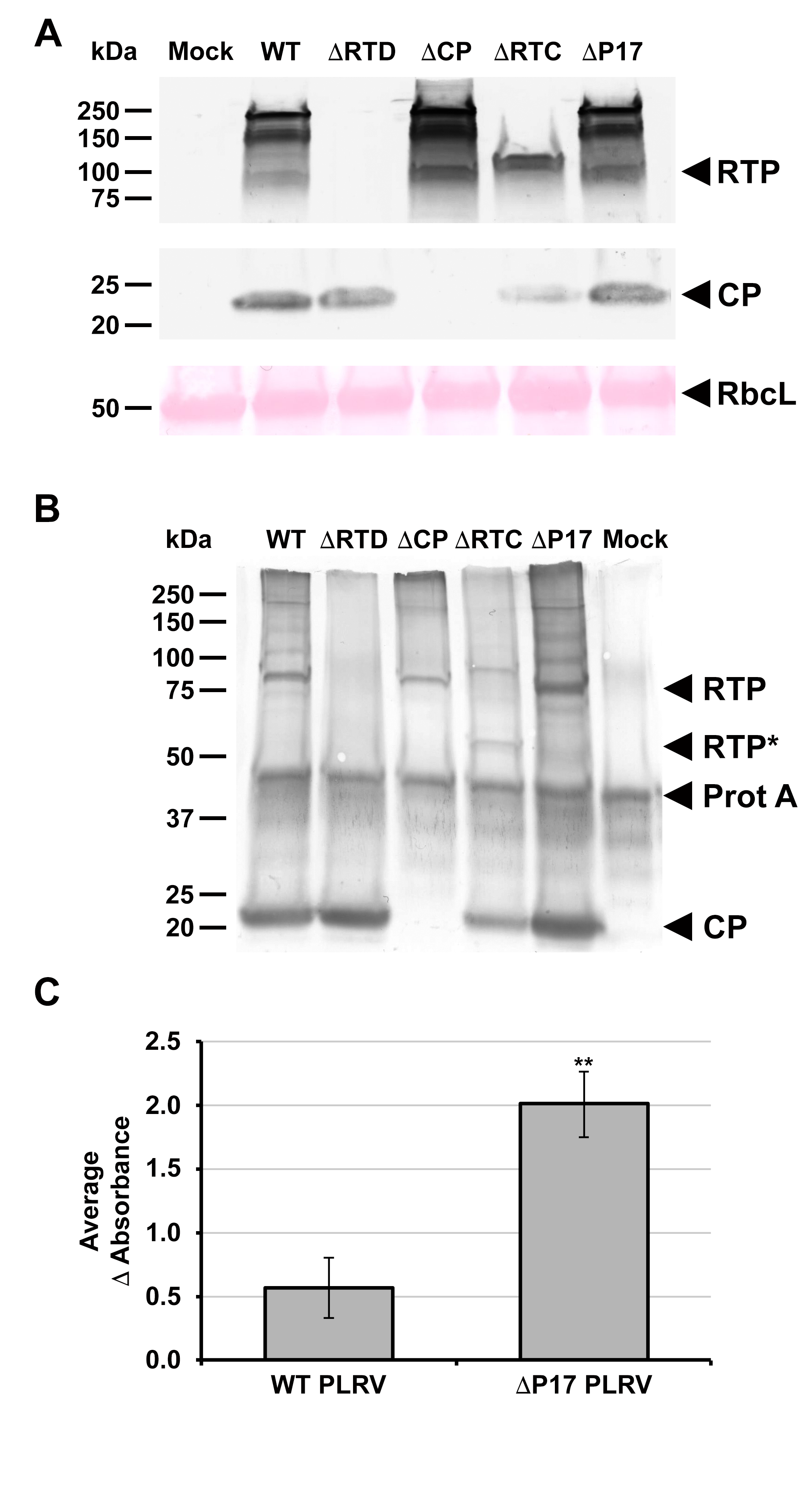

Supplement: Supplementary file 1 [file viruses-10-00585-s001.zip › 7.viruses-352914 suppl/DeBlasio_Viruses_FigureS2.tif]

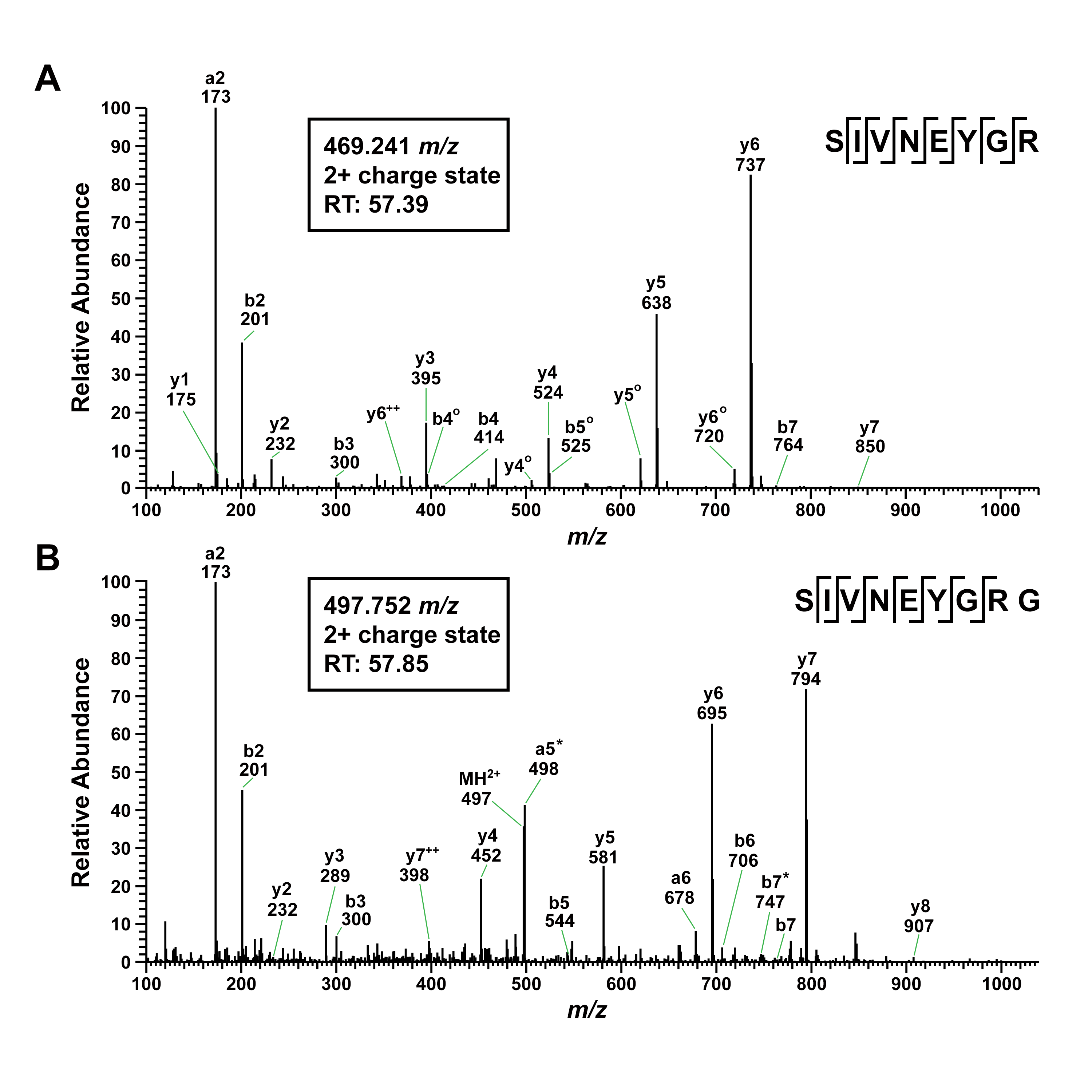

Supplement: Supplementary file 1 [file viruses-10-00585-s001.zip › 7.viruses-352914 suppl/DeBlasio_Viruses_FigureS3.tif]

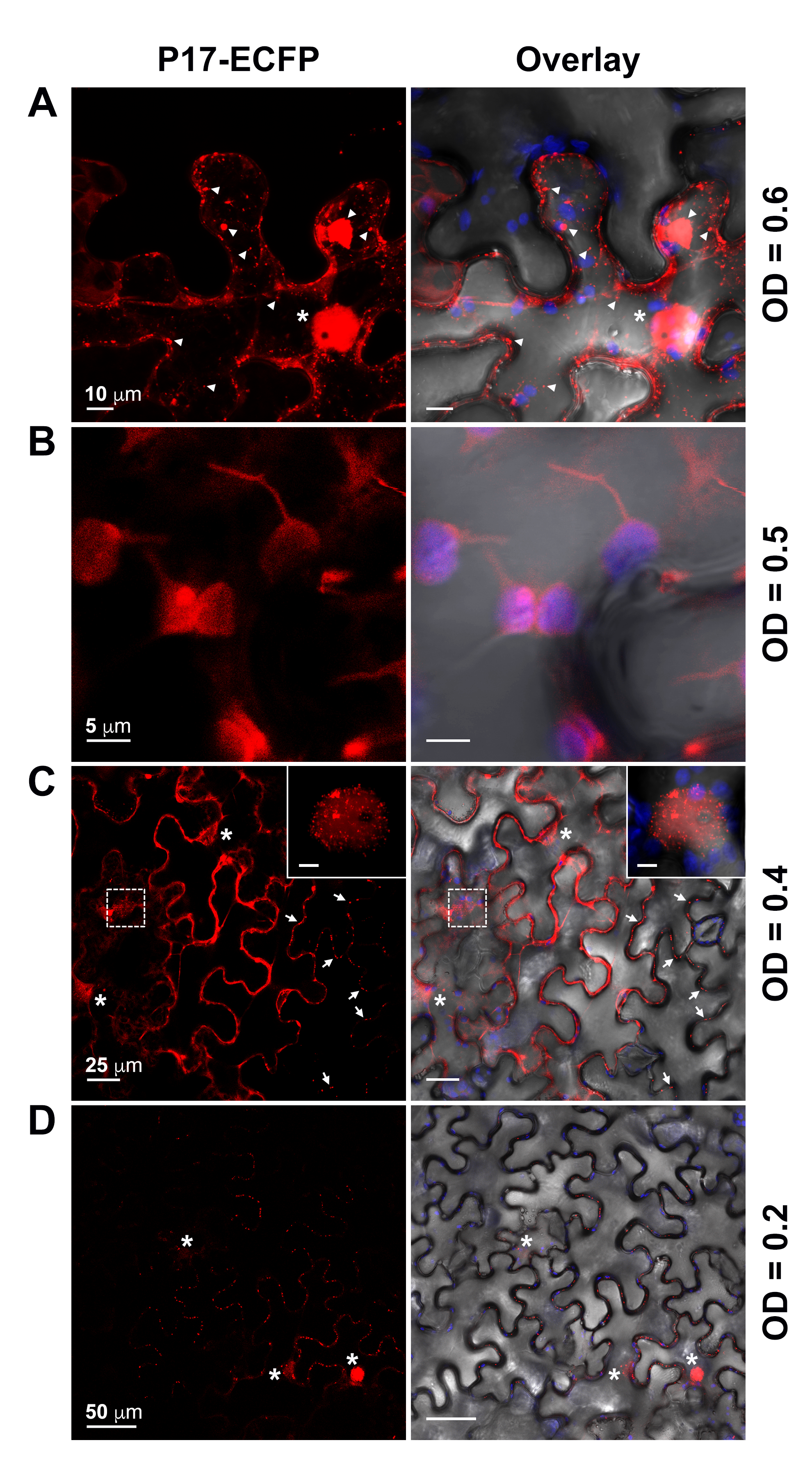

Supplement: Supplementary file 1 [file viruses-10-00585-s001.zip › 7.viruses-352914 suppl/DeBlasio_Viruses_FigureS4.tif]

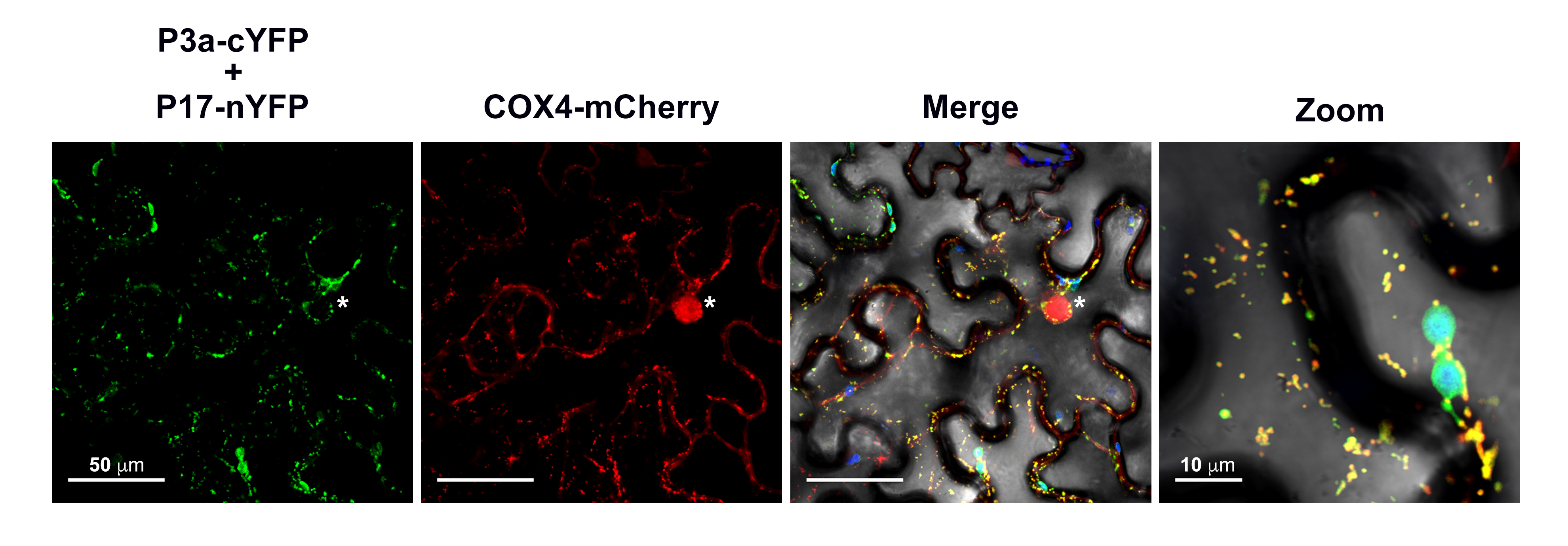

Supplement: Supplementary file 1 [file viruses-10-00585-s001.zip › 7.viruses-352914 suppl/DeBlasio_Viruses_FigureS6.tif]

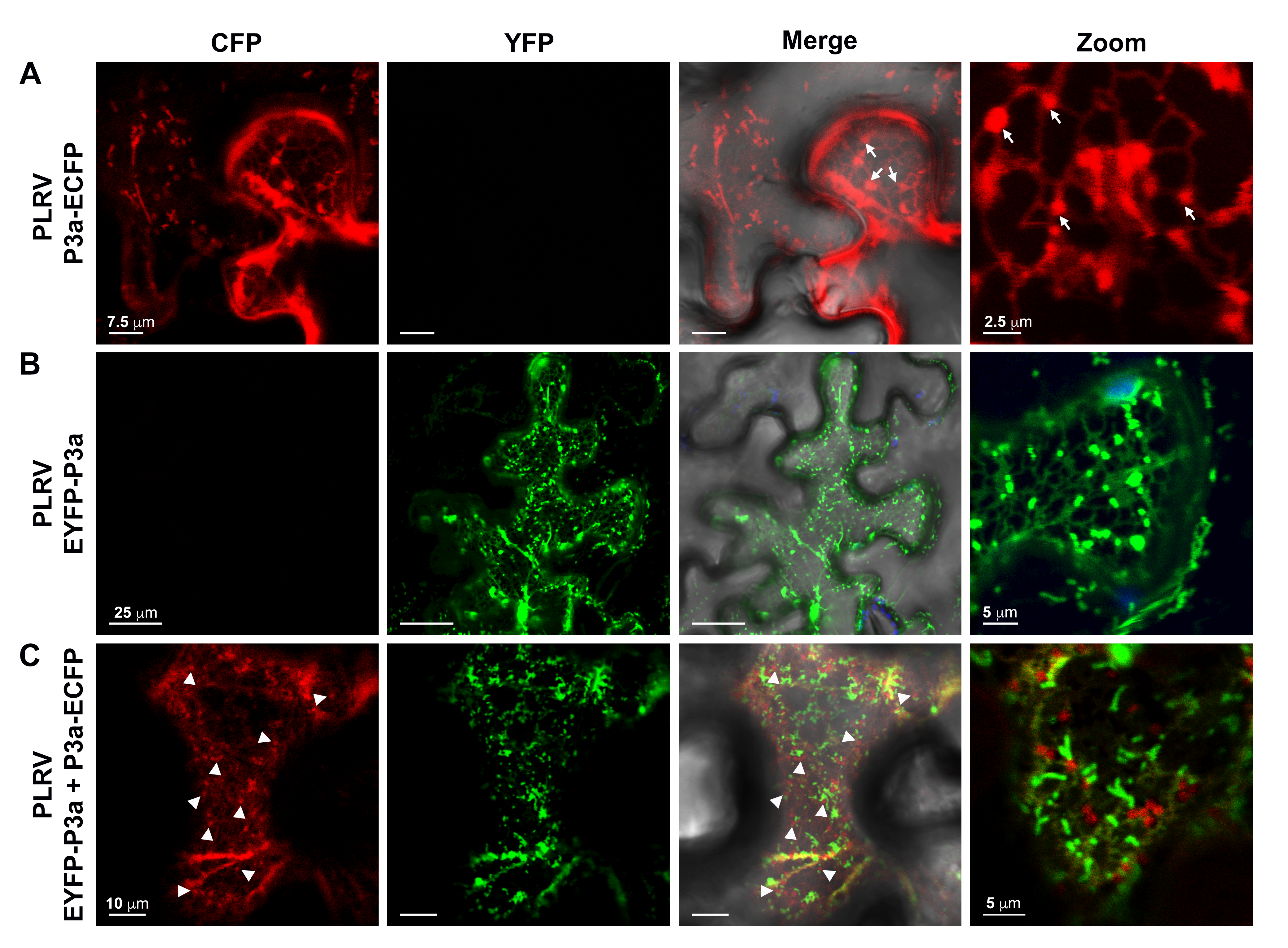

Supplement: Supplementary file 1 [file viruses-10-00585-s001.zip › 7.viruses-352914 suppl/DeBlasio_Viruses_FigureS7.tif]
